# Synthesis of Closely‐Contacted Cu_2_O‐CoWO_4_ Nanosheet Composites for Cuproptosis Therapy to Tumors With Sonodynamic and Photothermal Assistance

**DOI:** 10.1002/advs.202410621

**Published:** 2024-11-21

**Authors:** Zhuoran Yang, Zhuo Li, Chunyu Yang, Li Meng, Wei Guo, Liqiang Jing

**Affiliations:** ^1^ Key Laboratory of Photochemical Biomaterials and Energy Storage Materials Heilongjiang Province and College of Chemistry and Chemical Engineering Harbin Normal University Harbin 150025 China; ^2^ Key Laboratory of Functional Inorganic Materials Chemistry (Ministry of Education) School of Chemistry and Materials Science International Joint Research Center for Catalytic Technology Heilongjiang University Harbin 150080 China

**Keywords:** copper ion release, Cu_2_O‐CoWO_4_ nanocomposite, photothermal therapy, sonodynamic therapy, tumor therapy

## Abstract

Cuproptosis offers a promising and selective therapeutic strategy for cancer therapy. To fully realize its potential, the development of novel cuproptosis therapeutic agents and the achievement of efficient copper release are critical steps forward. Herein, closely‐contacted Cu_2_O‐CoWO_4_ nanosheet heterojunctions (CCW‐NH) are successfully synthesized using an in‐situ process for cuproptosis therapy. The efficient release of copper ions from CCW‐NH can be triggered by ultrasound irradiation, primarily due to the generation of superoxide radicals as the sonodynamic agents via the Z‐scheme charge transfer mechanism. When subjected to the combined effects of ultrasound and laser irradiation, the effective release of copper ions is increased by 1.7 times compared to the untreated group, significantly enhancing the efficiency of cuproptosis. And the incorporation of CCW‐NH and L‐arginine into the temperature‐sensitive injectable hydrogel (HP‐CCW@LA) ultimately achieved a tumor inhibition rate of up to 95.1%. L‐arginine, serving as a reducing agent, enabled the sustained release of highly active Cu^+^ during treatment. Notably, after treating tumors with HP‐CCW@LA, the tumor microenvironment is leveraged to promote copper ion conversion, which offers the potential for monitoring tumor therapy efficacy through magnetic resonance imaging. This work offers a novel integrated strategy for the development of new cuproptosis agents and therapeutic evaluation.

## Graphical abstract

Cu_2_O‐CoWO_4_ (CCW) nanosheet Z‐scheme heterojunctions possess sonodynamic and photothermal properties enabling efficient release of Cu^2+^ and demonstrating significant efficacy in tumor cuproptosis therapy. By incorporating CCW and L‐arginine into a temperature‐sensitive hydrogel, a high tumor inhibition rate of 95.1% is achieved, with potential for MRI‐based monitoring of treatment efficacy.

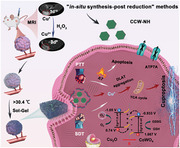

## 1 Introduction

Copper is an essential trace element central to biological processes, such as cellular respiration, iron metabolism, and connective tissue formation.^[^ ^1^ ^]^ Copper has a dual role in the tumor microenvironment. Tumor cells depend on copper for their normal cellular functions, making copper starvation a viable strategy to cause tumor cell death.^[^ ^2^ ^]^ However, excess copper can disrupt cellular homeostasis, leading to enzyme dysfunction, DNA damage, and a novel form of programmed cell death called cuproptosis.^[^ ^3^ ^]^ Cuproptosis affects the activity of lipoylation enzymes within the mitochondrial tricarboxylic acid (TCA) cycle, leading to the accumulation of acylated proteins, particularly dihydrolipoamide S‐acetyltransferase (DLAT) and iron‐sulfur cluster protein (FDX1) depletion.^[^ ^4^ ^]^ Interestingly, normal cells possess mechanisms that regulate copper levels and mitigate harmful consequences. Thus, cuproptosis could be a selective and effective therapeutic strategy for tumor cells.^[^ ^5^ ^]^ Moreover, Cu^+^ exerts more toxicity than Cu^2+^ toward tumor cells because of the direct binding of inactivated DLAT, forming inactive oligomers.^[^ ^6^ ^]^ Therefore, to achieve a more favorable cuproptosis effect, it is imperative to ensure the abundant presence of Cu^+^ ions during the treatment process. Thus, developing a highly efficient cuproptosis therapeutic approach to tumors is feasible and significant.

Recent advancements in nanotechnology have paved the way for developing efficient copper‐based nanotherapeutics that exploit cuproptosis for cancer therapy, such as self‐assembled nanoparticles, copper‐containing metal‐organic frameworks, and copper‐containing semiconductors.^[^ ^7^ ^]^ Ning et al. developed a smart cell‐derived nanorobot using copper‐doped zeolitic imidazolate framework‐8. This combines sonodynamic therapy (SDT) and cuproptosis for cancer treatment.^[^ ^8^ ^]^ The nanorobot achieved augmented tumor accumulation, triggered sonodynamic cuproptosis, and exhibited ultrasound (US)‐responsive cytotoxicity toward cancer cells. Xu et al. constructed a photothermally triggered nanoplatform (Au@MSN‐Cu/PEG/DSF) for synergistic cancer therapy via cuproptosis promotion.^[^ ^9^ ^]^ This platform facilitates on‐demand delivery of copper and disulfiram to cancer cells, forming cytotoxic bis(diethyldithiocarbamate)‐copper and Cu^+^ species and causing cell apoptosis and cuproptosis. The photothermal‐combined therapy significantly suppresses tumor growth with minimal toxicity, a promising strategy for targeted and effective cancer treatment. Naturally speculated, it is of great potential to develop sonodynamic and photothermal‐assisted novel cuproptosis therapeutic agents for tumors by synergistic antitumor effects. This is because copper release could be facilitated during SDT and photothermal therapy (PTT) processes, which are widely accepted.

Cu_2_O, as a narrow‐bandgap semiconductor, exhibits release copper with exceptional photothermal conversion ability, making it a promising candidates for cuproptosis treatment.^[^ ^10^ ^]^ However, its efficiency is often hindered by significant electron‐hole recombination, resulting in a low quantum yield and limited therapeutic effectiveness.^[^ ^11^ ^]^ To address this issue, constructing a Z‐scheme heterojunction with an oxide‐based semiconductor offers a viable strategy to enhance electron‐hole separation and improve photocatalytic performance. Furthermore, Z‐scheme heterojunctions offer three primary advantages: high charge separation efficiency, robust redox capability, and a broad catalytic reaction spectrum.^[^ ^12^ ^]^ These properties collectively position Z‐scheme heterojunctions as highly promising for biomedical applications. Given the critical role of tumor imaging during treatment, incorporating materials with strong computed tomography (CT) imaging capabilities and excellent biocompatibility is essential. Building on these considerations, CoWO_4_ could be employed to modulate Cu_2_O by forming Z‐scheme Cu_2_O‐CoWO_4_ heterojunctions, leveraging their complementary energy band structures. The photoexcited electrons from Cu_2_O could react with oxygen to generate superoxide radicals (·O_2_^−^), which could induce tumor cell apoptosis. Although Cu^+^ (3d^10^) lacks magnetic resonance imaging (MRI) capabilities, after the treatment of Cu_2_O‐CoWO_4_ nanosheet heterojunction (CCW‐NH) in specific tumor oxidative environments, Cu^+^ can be oxidized by endogenous hydrogen peroxide to Cu^2+^ (3d^9^), thereby endowing the system with MRI properties Thus, regulating the valence state of copper ions enables MRI to effectively monitor the therapeutic efficacy during treatment. There are limited reports on Cu_2_O heterojunctions with tungstates, including Cu_2_O‐BiWO_4_ and Cu_2_O‐CaWO_4_, and these studies predominantly focus on their photocatalytic performance in water oxidation and the degradation of organic pollutants.^[^ ^13^ ^]^ Notably, there is a lack of exploration regarding their potential applications in tumor therapy, particularly in relation to cuproptosis. Consequently, the development of this novel cuproptosis therapeutic agents (Cu_2_O‐CoWO_4_ heterojunctions), holds significant promise for advancing tumor therapy.

Due to the exceptionally large surface area and distinct physicochemical properties of 2D nanosheet materials, constructing a CCW‐NH holds great significance.^[^ ^14^ ^]^ These nanosheet structures could provide a larger contact area toward tumor cells while facilitating the rapid release of copper ions. However, heterogeneous structures face high interfacial transfer resistance, primarily due to interfacial binding problems and complex synthesis schemes.^[^ ^15^ ^]^ It is crucial to overcome the resistance through innovative synthesis techniques and interfacial engineering to realize the full potential of such promising nanosheet structures in biomedical applications. *In‐situ* strategies can provide a uniform distribution of components, synthesizing a heterogeneous compound with a closer interface.^[^ ^16^ ^]^ Nonetheless, direct CCW‐NH preparation using an *in‐situ* hydrothermal reaction strategy would disrupt the CoWO_4_ crystal formation under high temperature and pressure conditions with reducing agents, consequently impacting the heterojunction's structure and performance.^[^ ^17^ ^]^ We strategically propose to develop an “*in‐situ* synthesis‐post reduction” two‐step method for CCW‐NH to address the issue. First, a closely‐contacted nanocomposite heterojunction between CoWO_4_ and CuO is obtained using the same O element through the *in‐situ* synthesis.^[^ ^18^ ^]^ Second, NaBH_4_ is selected to reduce the obtained CCW‐NH at room temperature for controllably changing CuO to Cu_2_O by replacing with H_2_ employed reduction at high temperatures, simultaneously keeping the crystal structure and micro‐morphology of CoWO_4_.^[^ ^19^ ^]^ The uniform copper component distribution on the surface of CCW‐NH will significantly release copper ions and promote the cuproptosis effect in tumor cells. CCW‐NH would be mixed with a temperature‐sensitive hydrogel to enhance its applicability, maintaining an injectable sol state in vitro. Upon tumor injection, it would rapidly transform into a gel state, enabling CCW‐NH to accumulate at the tumor site and facilitating easier absorption by tumor cells. Furthermore, the incorporation of L‐arginine (L‐Arg) into the hydrogel system (HP‐CCW@LA) establishes a reductive therapeutic environment that enhances the sustained release of more reactive Cu^+^ ions from CCW‐NH, making it a crucial component in the treatment strategy. Meanwhile, L‐arginine can be decomposed into NO gas after the treatment process.^[^ ^20^ ^]^ Remarkably, following treatment with HP‐CCW@LA, the tumor microenvironment facilitates the conversion of Cu^+^ to Cu^2+^, enabling real‐time monitoring of treatment efficacy through MRI.

**Scheme 1:**
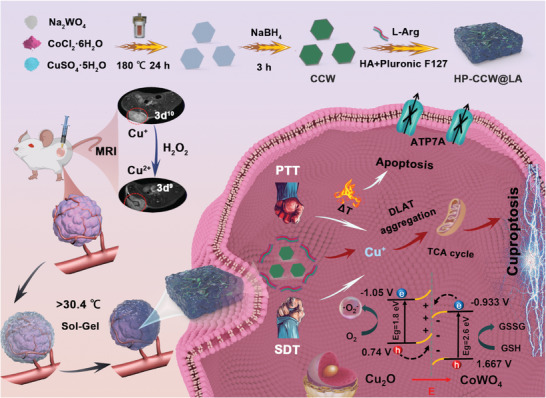
A schematic representation of the synthesis of an injectable hydrogel based on the CCW Z‐scheme heterostructure, along with a diagram illustrating the mechanism of tumor‐enhanced cuproptosis and multifunctional imaging capabilities.

This study successfully developed closely‐contacted CCW‐NH with a Z‐scheme electron transfer mechanism, which promotes the release of copper ions to enhance cuproptosis effects with the assistance of sonodynamic and photothermal effects (**Scheme** **1**). CCW‐NH was prepared using an *in‐situ* synthesis method, with precise control of the reaction time and NaBH_4_ reduction, to optimize its sonodynamic and photothermal performance. Furthermore, CCW‐NH was incorporated with L‐Arg into a temperature‐sensitive injectable hydrogel to effectively accumulate in the tumor region and achieve sustained release of more potent Cu^+^ ions, thereby maximizing therapeutic efficacy. Specifically, in the oxidative environment of tumors, Cu^+^ is oxidized by endogenous hydrogen peroxide to Cu^2+^ (3d^9^), while the appropriate addition of L‐Arg confers MRI properties to HP‐CCW@LA. Furthermore, HP‐CCW@LA also exhibits excellent CT and photoacoustic (PA) imaging capabilities. This study rationally designed and developed a novel cuproptosis therapeutic agent aimed at improving the efficacy of tumor treatment and provides valuable insights for advancing the new generation of copper‐based materials for integrated treatment and diagnosis strategies.

## 2 Results and Discussion

### 2.1 Synthesis and Characterization of CCW‐NH

**Figure 1:**
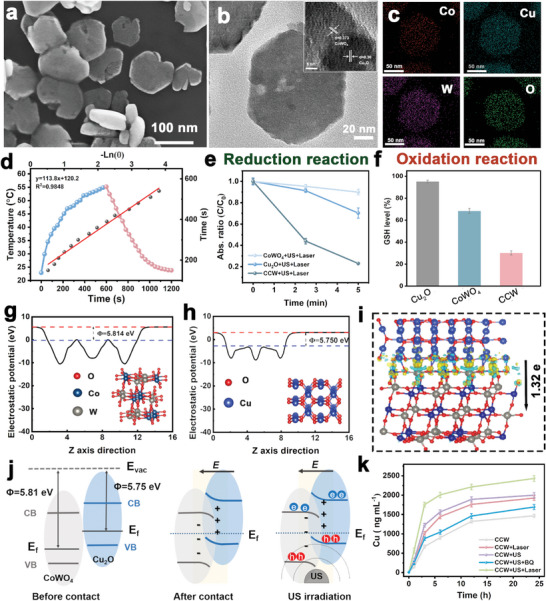
a) Scanning electron microscope (SEM) and b) Transmission electron microscopy (TEM) images of CCW‐NH. Inset: the HRTEM image. c) TEM‐EDS mapping of CCW, including Co, Cu, W, and O elements. d) Heating and cooling curves for CCW. The cooling time was plotted against the negative natural logarithm of the temperature driving force. e) The detection of ·O_2_^−^ using NBT probes. f) GSH consumption using Cu_2_O, CoWO_4_ and CCW under US irradiation. g,h) The calculated work functions of CoWO_4_ (‐1 1 0) and Cu_2_O (1 1 0) surfaces. i) The 3D charge density difference in the CCW‐NH reveals variations between CoWO_4_ and Cu_2_O. The blue and yellow regions denote charge depletion and accumulation. j) A schematic illustration of the directional charge transfer process in the CoWO_4_/Cu_2_O interface. It was affected by the interfacial electric field and US irradiation. k) The release of copper ions from CCW‐NH dispersions under US, laser, or US+laser irradiation. Data are presented as the mean ± SD (n = 3).

Initially, we varied the precursor reaction temperature within the range of 160 °C and 200 °C. The synthesized precursors demonstrated negligible differences in crystal composition; however, at excessively low temperatures, they exhibited uneven particle sizes, whereas excessively high temperatures led to agglomeration. Therefore, 180 °C identified as the optimal reaction temperature (Figures S1–S3, Supporting Information). Subsequently, we adjusted the concentration of the copper source from 0 to 1.2 mmol. X‐ray diffraction (XRD) analysis revealed that the crystal composition of these samples exhibited minimal variation; however, an excess of copper ions adversely affected the morphology of the precursors. Consequently, under the condition of maximizing copper content, a concentration of 0.6 mmol was deemed optimal (Figures S4–S6, Supporting Information). Additionally, we adjusted the hydrothermal reaction time. Although the reaction time had a minimal effect on the crystal composition of the precursors, excessively short reaction durations resulted in uneven particle sizes, while extended reaction times caused agglomeration. Thus, a reaction duration of 24 h was concluded to be optimal (Figures S7–S9, Supporting Information). The optimized precursor showed a uniform nanosheet structure, with an average particle size of nearly 106 nm and a thickness of ≈27 nm (Figures S10 and S11, Supporting Information). Subsequently, Cu^2+^ ions in the precursor were reduced using NaBH_4_ to obtain the CCW‐NH, with a reduction time ranging between 1 and 6 h (**Figure** **1**a; Figure S13, Supporting Information). XRD analysis depicted the coexistence of Cu_2_O and CoWO_4_ crystalline phases (Figure S14, Supporting Information). Moreover, X‐ray photoelectron specroscopy (XPS) helped determine the valence states of Co, Cu, and W in CCW‐NH (Figures S15–S17, Supporting Information). The peaks in the XPS spectra at 796.7 eV and 780.8 eV were assigned to the Co^2+^ spin‐orbit coupling.^[^ ^21^ ^]^ For the Cu 2p spectrum, binding energies at 952.5 eV and 932.5 eV corresponded to Cu^+^, while 953.9 eV and 934.5 eV corresponded to Cu^2+^, depicting partial oxidation at the CCW‐NH surface.^[^ ^22^ ^]^ The binding energies in the W 4f spectrum at 37.4 eV and 35.3 eV were assigned to W^6+^.^[^ ^23^ ^]^ Compared to CCW‐NH, the precursor lacked Cu^+^, and the Co and W valence states were unchanged after reduction treatment (Figure S12, Supporting Information). The above results suggested that using the mild reducing agent NaBH_4_ ensures Cu^2+^ and avoids W^6+^ reduction, reflecting the “in situ synthesis‐post‐reduction” advantage. Then, the effect of different reduction times was studied on the optical absorption of CCW‐NH. The three reduced samples exhibited improved optical absorption compared to the precursors because of the Cu_2_O formation (Figure S18, Supporting Information). However, the CCW dispersion absorbance decreased when the reduction time was extended to 6 h. This could be due to prolonged stirring leading to peroxidation of the CCW‐NH surface. Additionally, photocurrent data showed no significant elevation in charge transfer between Cu_2_O and CoWO_4_ interfaces after 3 h compared to 6 h (Figure S19, Supporting Information). Therefore, CCW‐NH‐based samples were selected with a reduction time of 3 h for further investigation. The resulting CCW‐NH maintained a uniform morphology without significant changes observed in particle size or thickness (Figure S20, Supporting Information). A high‐resolution transmission electron microscopy image (HRTEM) depicted the formation of a heterogeneous structure to clarify the relationship between Cu_2_O and CoWO_4_. The (−1 1 0) crystal plane of CoWO_4_ interfaced with the (1 1 0) crystal plane of Cu_2_O (Figure 1b). TEM‐energy dispersive spectrometer (EDS) mapping validated the uniform dispersion of Co, Cu, W, and O elements in CCW‐NH (Figure 1c). The uniformly dispersed nanosheets of this element help reduce the interfacial resistance between the heterogeneous structures and contribute to rapidly releasing copper ions, thus improving the cuproptosis effect. Next, the temperature response of CCW‐NH suspension was investigated when exposed to laser radiation at 655 nm (Figure S21). Pure water experienced an 8.5 °C temperature increase after 600 s laser irradiation. In contrast, CCW‐NH exhibited a temperature increase of 41.0 °C at 1 mg mL^−1^ under identical conditions, demonstrating exceptional photothermal conversion capability. Moreover, Figure S21b (Supporting Information) depicts a positive correlation between laser irradiation power density and the temperature elevation of CCW‐NH. Temperature increase and decrease tests were performed on the CCW‐NH dispersion, and the photothermal conversion efficiency of CCW was 24.51% (Figure 1d). Five cycles of heating and cooling curves were performed on CCW dispersion, demonstrating its significant photothermal stability (Figure S21c, Supporting Information). The p‐Nitro‐Blue tetrazolium chloride (NBT) probe helped detect ·O_2_^−^ formation using the ultraviolet‐visible (UV–vis) absorption method. Under US irradiation, the amount of ·O_2_^−^ produced using CCW was significantly higher than the CoWO_4_ and Cu_2_O groups (Figure 1e). At 5 min, the amount of ·O_2_^−^ synthesized by CCW was 7.7 times that of CoWO_4_ and 2.8 times that of Cu_2_O. The abundant generation of ·O_2_^−^ not only facilitates the release of copper ions but also disrupts ATP7A on the cell membrane surface, thereby inhibiting the efflux of copper ions and ultimately enhancing the cuproptosis effect. Compared to the control groups (water and CCW), CCW combined with laser or US generated abundant ·O_2_^−^ (Figure S22, Supporting Information). Under laser irradiation, CCW can synthesize ·O_2_^−^ using a 655 nm laser to excite Cu_2_O (Eg = 1.8 eV). CCW combined with both laser (655 nm) and US generated even more ·O_2_^−^. We employed the electron spin resonance (ESR) method using 5,5‐dimethyl‐1‐pyrroline N‐oxide as a trapping agent to detect ·O_2_^−^ (Figure S23, Supporting Information). ESR spectroscopy supported the above conclusion. Then, a glutathione (GSH) assay kit helped detect the reduced GSH levels. The results indicated that GSH consumption by CCW‐NH was more significant than Cu_2_O and CoWO_4_ alone (Figure 1f). As shown in Figure S24 (Supporting Information), the decreased GSH levels across different groups were consistent with the ·O_2_^−^ generation trend. The band structure with band gaps, conduction band (CB) levels, valence band (VB) levels, and electrostatic potentials, including work functions and Fermi levels, helped better understand the CCW‐mediated catalytic mechanism. We analyzed their UV–vis diffuse reflectance spectra and Mott–Schottky (MS) plots to determine the valence and conduction band positions of CoWO_4_ and Cu_2_O in the CCW heterojunction. As shown in Figure S25 (Supporting Information), CoWO_4_ showed valence and conduction bands at 1.67 V and −0.93 V, respectively, while Cu_2_O indicated valence and conduction bands at 0.75 V and −1.05 V, respectively. Moreover, the work function is an essential parameter reflecting the field‐emission properties of materials.^[^ ^24^ ^]^

Density Functional Theory (DFT) depicted work functions of 5.81 eV for CoWO_4_ and 5.75 eV for Cu_2_O, establishing the existence of charge migration at their interfaces (Figure 1g,h). The Bader charge analysis suggested that the charge transfer between Cu_2_O and CoWO_4_ interfaces is nearly 1.32 e (Figure 1i). The proposed formation mechanism of a Z‐scheme heterostructure (Figure 1j) involves electrostatic interactions inducing interfacial band bending and establishing a directional built‐in electric field at the Cu_2_O to CoWO_4_ heterojunction interface. Electrons in the conduction band of CoWO_4_ combine with holes in the valence band of Cu_2_O driven by the built‐in electric field and the US irradiation. This leads to electrons with high reduction potential in the Cu_2_O conduction band and holes with high oxidation potential in the CoWO_4_ valence band. The CCW‐NH dispersion is capable of releasing copper ions, and both SDT and PTT can facilitate this release process. Compared to the untreated group, the copper ion release from the CCW‐NH dispersion, when combined with US or laser irradiation, was respectively enhanced by 1.4 and 1.3 times. Notably, under the simultaneous application of US and laser irradiation, the released copper ions could be boosted up to 1.7 times, which is conducive to augmenting the cuproptosis effect of CCW‐NH (Figure 1k). The massive release of ·O_2_^−^ can affect the stability of the nanomaterial surface, leading to surface ion exposure and subsequent release into the solution. To substantiate this hypothesis, we conducted additional control experiments using p‐benzoquinone (BQ) as a scavenger of superoxide radicals and examined the release of copper ions from CCW under ultrasonic conditions (Figure 1k). Compared with the control group without the scavenger, we observed a noticeable reduction in copper ion release in the presence of BQ, indicating that while the cavitation effect of ultrasound alone contributes to copper ion release, superoxide radicals significantly enhance this process. This finding further validates our proposed mechanism. We further evaluated the stability of CCW‐NH under different simulated physiological conditions, including simulated buffer solutions, serum, and culture medium, and determined the copper ion release curves under different conditions. As shown in Figure S26 (Supporting Information), copper ions were released from Cu_2_O‐CoWO_4_ nanosheets under all three simulated physiological conditions, and the release rate of copper ions did not show significant differences, with slightly slower release in fetal bovine serum (FBS). In addition, the released copper ions can catalyze the decomposition of H_2_O_2_ at the tumor site, generating hydroxyl radicals and oxygen, thereby ensuring an adequate oxygen supply throughout the treatment. To substantiate this, we conducted additional experiments. The results demonstrated that, compared to the control group, CCW can efficiently catalyze the production of oxygen from H_2_O_2_ (Figure S27, Supporting Information).

### 2.2 Cuproptosis‐Inducing Efficacy and Mechanism of CCW on Cancer Cells under Combined Therapies

**Figure 2:**
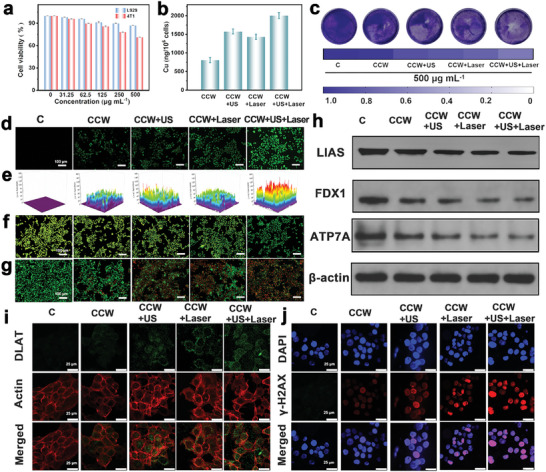
a) CCW cytotoxicity assessment on L929 and 4T1 cells at different concentrations with the MTT assay. b) Copper release in CCW co‐culture with 4T1 cells is monitored under different treatments. c) The plate clone formation assay of CCW‐treated groups after different treatments. d) ROS generation measurement across distinct experimental groups using the DCFH‐DA probe. e) Quantitative analysis of ROS fluorescence intensity within the respective groups. f) The assessment of cell membrane permeability across different groups using the AO dye. g) The evaluation of cell viability in different experimental groups through Ca‐AM/PI staining. h) Protein expression examination across various groups through western blot analysis. i) Visualizing DLAT oligomerization in varied experimental groups with immunofluorescence imaging. j) The DNA damage detection in different experimental groups. Data are presented as the mean ± SD (n = 5).

The cytotoxic effects of CCW on L929 (normal) and 4T1 (cancer) cells were evaluated using the thiazolyl blue tetrazolium bromide (MTT) assay. At a CCW concentration of 500 µg mL^−1^, the cell viability of L929 and 4T1 cells was 87% and 71%, respectively. Therefore, CCW exhibits low cytotoxicity toward normal cells. However, it exerts a pronounced toxic effect on 4T1 cancer cells, potentially via the cuproptosis effect (**Figure** **2**a). Subsequently, CCW was co‐cultured using 4T1 cells, and the intracellular copper concentration was monitored over different time intervals with inductively coupled plasma mass spectrometry (ICP‐MS), as depicted in Figure S28 (Supporting Information). We also observed that US or laser irradiation can enhance the release of copper ions inside cells. The combination of US and laser irradiation can release more copper ions, indicating that the synergistic effect of photothermal and sonodynamic therapies can enhance the cuproptosis effect (Figure 2b). The results revealed a progressive elevation in copper concentration with prolonged culture time. Thus, the sustained release of copper ions using CCW into the cells is a key cellular cuproptosis factor. Intracellular levels of reactive oxygen species (ROS) were evaluated using 2,7‐dichlorodihydrofluorescein diacetate (DCFH‐DA). Intracellular levels of ·O_2_^−^ production were consistent with the in vitro results (Figure 2d,e). Therefore, we assessed the membrane permeability across different treatment groups (Figure 2f). The membranes of normal cells were stained yellowish‐green using acridine orange hydrochloride (AO). Alterations in membrane permeability penetrated AO into the cells and its binding to DNA, synthesizing green fluorescence. There was a positive correlation between ·O_2_^−^ generation and variations in membrane permeability across the different groups. Plate clone formation assays helped assess cell survival after different treatments (Figure 2c). Compared to the control group, the CCW‐treated group showed a diminished number of cell clones, suggesting cuproptosis as the underlying mechanism. Furthermore, the CCW+US and CCW+Laser groups depicted even fewer cell clones, signifying that the co‐administration of CCW with either US or laser augmentation enhanced cuproptosis. Combining CCW‐NH with US and laser led to the lowest cell clone counts, improving the cuproptosis effects. Cell viability across different treatment groups was assessed with calceinacetoxymethyl/propidium iodide (Ca‐AM/PI) staining. The staining outcomes aligned with the trends within cell death observed in the plate clone formation experiment (Figure 2g).

Cell apoptosis in various experimental groups was quantified via flow cytometry analysis (Figure S29, Supporting Information). Compared to the control group, the CCW‐treated group depicted an apoptosis rate of 23.3%, predominantly caused by cuproptosis. Remarkably, the CCW+US+Laser group showed a substantially elevated apoptosis rate of 71.4%, surpassing the rates observed in the CCW+US (33.5%) and CCW+Laser groups (45.0%). Moreover, the decline in intracellular ATP levels and the elevation of JC‐1 monomers align with the severity of 4T1 copper‐induced cell death (Figures S30 and S31, Supporting Information). We substantiated our prior hypothesis by assessing the expression of cuproptosis‐associated proteins, viz., LIAS, FDX1, and ATP7A, using western blot analysis and employed immunofluorescence to detect DLAT oligomerization (Figure 2h). The primary ATP7A protein function is to extrude excess copper ions from cells, and its activity diminishes upon copper‐induced cell death, leading to decreased expression.^[^ ^25^ ^]^ Tumor cells have a complex regulatory system to control copper uptake, excretion, and distribution. During copper overload, copper ions are transported from the trans‐Golgi network to the cell membrane for export by Cu‐ATPase (such as ATP7A). Superoxide radicals are potent oxidants that can induce mitochondrial dysfunction, thereby disrupting the energy supply to efflux pathways. Additionally, these radicals can directly oxidize critical amino acid residues in the ATP7A protein, such as cysteine and tyrosine. This oxidative modification can lead to alterations in the protein's structure and function. Consequently, the oxidative damage may disrupt the 3D conformation of ATP7A, thereby impairing its copper ion transport capabilities.^[^ ^26^ ^]^ FDX1 and LIAS have been shown to be key effectors of lipid acylation and play a critical role in the subsequent initiation of cellular cuproptosis.^[^ ^27^ ^]^ The CCW+US+Laser group depicted the lowest levels of LIAS, FDX1, and ATP7A expression compared to the other groups. Thus, CCW combined with US or laser can improve the cuproptosis effect, with the most significant enhancement seen when CCW was administered with US and laser. Dysregulated expression and functions of these proteins can cause an imbalance in copper ion metabolism, culminating in cuproptosis. DLAT is essential for the reduction of copper and causes aggregation upon binding to copper.^[^ ^28^ ^]^ DLAT oligomerization was detected using green fluorescence, while the cell cytoskeleton labeled with phalloidin showed red fluorescence (Figure 2i). No significant DLAT oligomerization was observed in the control group. However, the group treated with CCW‐NH caused DLAT oligomerization. DLAT oligomerization was notably improved when CCW was combined with US or laser treatment. This was because of the augmented effect of SDT and PTT facilitated by the CCW‐NH Z‐scheme heterojunction on cuproptosis. DLAT oligomerization was intensified when CCW‐NH was combined with US and laser irradiation, indicating a more substantial impact on cell cuproptosis. We hypothesize that cuproptosis may induce DNA damage in tumor cells. Immunofluorescence staining was performed on 4T1 cells to substantiate this hypothesis. The level of γ‐H2AX was significantly increased in DNA‐damaged tumor cells. Compared with the control group, cells in the CCW‐induced group showed DNA damage in the cell nucleus, primarily attributed to cuproptosis (Figure 2j).

### 2.3 Development of a Temperature Sensitive Hydrogel for Enhanced CCW‐NH Accumulation and Tumor Therapy

**Figure 3:**
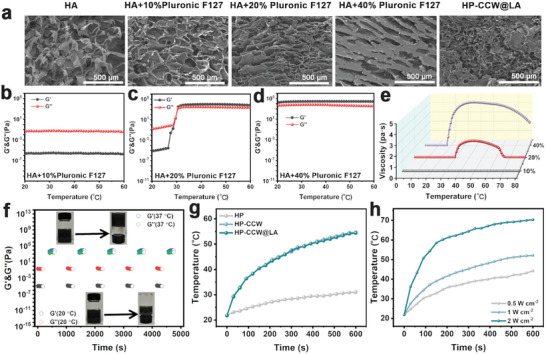
a) SEM images of different freeze‐dried samples. b‐d) Storage and loss moduli, and e) viscosity of the HA+10% Pluronic F127, HA+20% Pluronic F127, and HA+40% Pluronic F127. f) Sol‐gel transition performance test of HP‐CCW@LA across different temperatures. g) The photothermal curves of different samples. h) The photothermal curves of HP‐CCW@LA recorded under laser irradiation with different power densities.

CCW was incorporated with L‐Arg into a temperature‐sensitive hydrogel of Pluronic F127 and HA to enhance its applicability. **Figure** **3**a illustrates that the lyophilized HA sample exhibits a porous structure. The porosity increases significantly after adding 10% Pluronic F127 because of hydrogen bonding. Further, increasing the concentration of Pluronic F127 to 20% reinforces the porous structure. However, excessive concentration at 40% can destroy the porous structure. Rheological tests depict that the sample remains in solution form at temperatures ranging between 20 and 60 °C when using 10% Pluronic F127 (Figure 3b). However, a solution‐to‐gel transition occurs ≈31 °C at a 20% concentration (Figure 3c). This hydrogel remains in solution form in vitro, ensuring injectability, and transforms into a gel state upon injection within the tumor, facilitating CCW accumulation. The sample maintains a gel state between 20 and 60 °C as the concentration of Pluronic F127 increases (Figure 3d). Viscosity tests are consistent with those described above (Figure 3e). Adding CCW and L‐Arg does not impact the temperature sensitivity or rheological properties of the hydrogel (Figure S32, Supporting Information). Combining the above results, we selected the 20% Pluronic F127 sample for additional studies, exhibiting excellent solution‐to‐gel transition behavior (Figure 3f). HP‐CCW@LA demonstrates photothermal properties similar to CCW‐NH under 655 nm laser irradiation (Figure 3g), and its heating capability intensifies with enhancing laser power density (Figure 3h). In aqueous solution, Cu⁺ demonstrates a stronger catalytic ability for H_2_O_2_ compared to Cu^2+^.^[^ ^29^ ^]^ Therefore, by introducing 3,3',5,5'‐tetramethylbenzidine (TMB) probe and H_2_O_2_, we can indirectly prove that L‐Arg promotes the release of highly active Cu^+^ during the treatment process. We detected the generation of hydroxyl radicals by H_2_O_2_ catalyzed by copper ions released from CCW‐NH with or without L‐Arg. As illustrated, the presence of L‐Arg significantly boosts the catalytic efficiency of CCW in decomposing H_2_O_2_, leading to a higher production of hydroxyl radicals, which indicates the abundant presence of Cu⁺ (Figure S33, Supporting Information). In addition, the oxidative decomposition of L‐Arg produces NO, a small gaseous molecule with remarkable anti‐tumor properties.

### 2.4 In Vivo Therapeutic Efficacy of HP‐CCW@LA and its Potential for Monitoring Tumor Therapy

**Figure 4:**
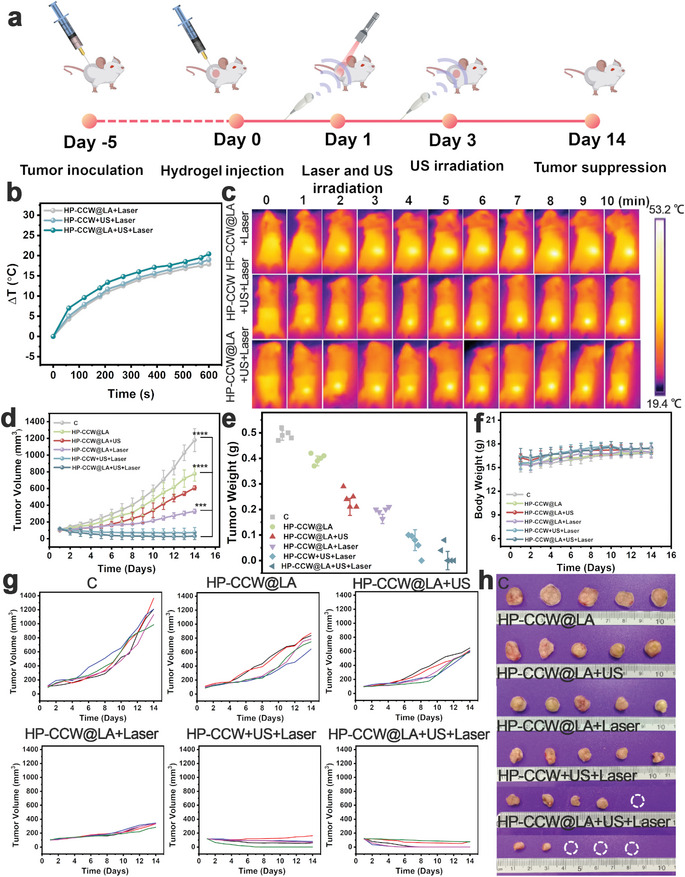
a) The treatment protocol for 4T1 tumor‐bearing mice. b) The tumor surface temperature curves and c) associated thermal images of the HP‐CCW@LA+Laser, HP‐CCW+US+Laser, and HP‐CCW@LA+US+Laser groups. d,g) Tumor volume (n = 5, ***P < 0.001, and ****P < 0.0001), e) tumor weight, f) body weight, and h) tumor photographs for the control and treatment groups. Data are presented as the mean ± SD (n = 5).

The therapeutic effect of the CCW‐NH‐based injectable hydrogel was further evaluated in vivo. 4T1 tumor‐bearing mice were randomly divided across six groups (n = 5), such as the control, HP‐CCW@LA, HP‐CCW@LA+US, HP‐CCW@LA+Laser, HP‐CCW+US+Laser, and HP‐CCW@LA+US+Laser groups. The corresponding treatment protocol is shown in **Figure** **4**a. Three groups underwent PTT, and an infrared camera helped monitor tumor temperature changes during treatment. While irradiating with a 655 nm laser (1 W cm^−2^), the temperature elevation on the surface of the tumors in the HP‐CCW@LA+Laser, HP‐CCW+US+Laser, and HP‐CCW@LA+US+Laser groups were similar. There was an increase of nearly 20 °C, depicting that the surface temperature increase was primarily induced by the photothermal effect (Figure 4b,c). After 14 days, the average tumor volume within the control group reached ≈1200 mm^3^. In contrast, it decreased to ≈800 mm^3^ in the HP‐CCW@LA group, primarily due to tumor cell cuproptosis (Figure 4d,g). Conversely, the average tumor volumes within the HP‐CCW@LA+US and HP‐CCW@LA+Laser groups were 600 and 300 mm^3^, respectively, suggesting enhanced cuproptosis effects with SDT or PTT. The combination of L‐Arg with SDT and PTT achieved the best therapeutic effect, with a tumor inhibition rate of up to 95.1% by day 14 (Figure 4h; Figure S34, Supporting Information). After the treatment, the tumors were prepared as cell suspensions and subsequently disrupted using a cell homogenizer. Following this, centrifugation was performed to remove undigested components, such as CCW‐NH and cell membranes. The copper ion concentrations were then assessed following treatment with various experimental groups. The results showed that the copper ion levels in the tumors of mice in the HP‐CCW@LA+US+Laser group were significantly higher than in the other experimental groups, proving that with the assistance of sonodynamic and photothermal effects, more copper ions are released, which is more conducive to cuproptosis therapy (Figure S35, Supporting Information). This suggests that CCW can effectively release copper ions under the assistance of sonodynamic and photothermal effects, especially in the presence of L‐Arg, which ensures the presence of Cu^+^ with high lethality, thereby achieving satisfactory tumor treatment outcomes. The trend of tumor volume data in different experimental groups was consistent with tumor size data in mice (Figure 4e). Based on the results of post‐treatment tumor weight in different groups and the Bliss Independence Model, there is a synergistic effect between sonodynamic and photothermal therapy. To further highlight the superior performance of Cu_2_O‐CoWO_4_ heterojunctions, we conducted a comparative study with chemotherapy and selected doxorubicin hydrochloride as a standard antitumor drug. Doxorubicin hydrochloride was incorporated into the prepared thermosensitive hydrogel, ensuring that its concentration was consistent with that of CCW. The tumor size results demonstrated that our novel approach significantly outperformed traditional chemotherapy (Figure S36, Supporting Information). Throughout the 14‐day treatment process, no significant decrease in body weight could be observed in the other groups compared to the control group, highlighting the safety of HP‐CCW@LA injectable hydrogel (Figure 4f). We measured the changes in W element content in the main organs and tumor sites of mice after intratumoral injection of HP‐CCW@LA using ICP‐MS. As shown in Figure S37 (Supporting Information), due to the intratumoral injection, the order of W element content in tumors and main organs is as follows: tumor > liver > kidney > spleen > lung > heart. Additionally, from day 1 to day 14, the content of tungsten gradually decreased. Furthermore, W element was primarily distributed in the liver, likely due to the filtering function of the mouse reticuloendothelial system, suggesting that CCW is mainly metabolized by the liver. The variations in the concentration of element W in the bloodstream indicate that CCW is capable of circulating within the blood. Notably, the concentration peaked at 12 h post‐administration, after which it gradually decreased (Figure S38, Supporting Information). As shown in Figure S39 (Supporting Information), the content of tungsten in mouse urine gradually decreased over 14 days, indicating that CCW can be excreted from the body through the urine. Hemolysis assay and H&E staining results of the main organs in mice further validated its good biocompatibility (Figures S40and S41, Supporting Information). Moreover, we continue to examine blood chemical analysis and blood routine tests of mice to evaluate the biocompatibility of the material. In the blood chemistry analysis, we carefully selected alkaline phosphatase (ALP), aspartate aminotransferase (AST), and alanine aminotransferase (ALT) as indicators of liver function (Figure S42, Supporting Information). After 14 days of treatment, all of these test indicators remained within normal ranges in different groups, demonstrating that HP‐CCW@LA did not cause significant liver toxicity. Furthermore, we also evaluated the blood routine of mice after 14 days, including white blood cells (WBC), red blood cells (RBC), platelets (PLT), mean corpuscular hemoglobin (MCH), mean corpuscular volume (MCV), hematocrit (HCT), hemoglobin (HGB), and mean corpuscular hemoglobin concentration (MCHC) as key indicators (Figure S43, Supporting Information). Compared to the control group, there were no significant changes in different blood routine indicators in the mice of the different treatment groups, further confirming the good biocompatibility of HP‐CCW@LA. In addition to the initial two‐week treatment period, we conducted an additional four‐week follow‐up study on the mice and plotted the survival curves to evaluate the combined effect of HP‐CCW@LA with ultrasound and laser irradiation (Figure S44, Supporting Information). The results showed that no mice in the HP‐CCW@LA +US+Laser group experienced mortality, which is highly satisfactory.

**Figure 5:**
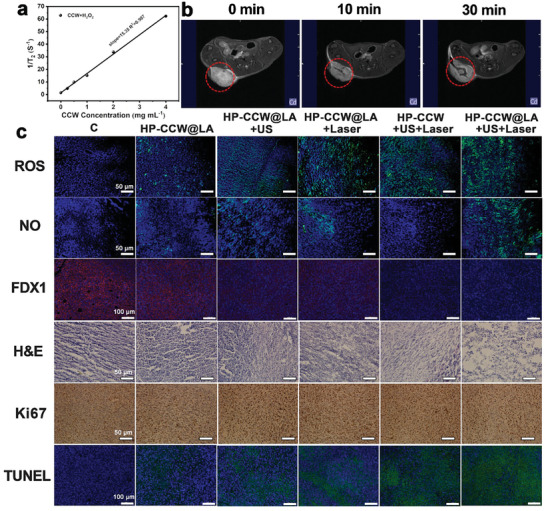
a) The relationship curve between concentration and 1/T_2_ for CCW+H_2_O_2_ (10 mM). b) The MRI effects of mouse tumor sites at different time intervals after HP‐CCW@LA injection. c) ROS, NO, FDX1, H&E, Ki67, and TUNEL staining images of mice tumor sections after undergoing different treatments.

Monitoring tumors after treatment is crucial. However, CCW is primarily composed of Cu^+^, which lacks MRI imaging capability owing to its 3d^10^ electron configuration. Meanwhile, L‐arginine aids in maintaining the valence state of Cu^+^. Notably, L‐arginine decomposes into NO after treatment, whereas CCW interacts with endogenous hydrogen peroxide at the tumor site, oxidizing Cu^+^ on its surface to Cu^2+^. This valence transition enables the use of CCW for MRI of tumors. As shown in **Figures** **5**a and S45 (Supporting Information), the transverse relaxivity (r2) value of CCW increases to 15.4 L g^−1^ s^−1^ after adding hydrogen peroxide. Following the injection of the HP‐CCW@LA hydrogel, the imaging effect is not significant in the initial stage, due to the unchanged valence state of Cu^+^. After 10 min, a portion of the Cu^+^ transformed into Cu^2+^, resulting in a slight imaging effect. Notably, after 30 min, the reaction with endogenous tumor hydrogen peroxide significantly enhances the T2 MRI signal (Figure 5b), ultimately resulting in a pronounced T2 imaging effect.

Due to the presence of W element (atomic number = 73) in the CCW, it could have an excellent capability for CT imaging.^[^ ^30^ ^]^ A standard curve was established and compared with iohexol using a micro‐CT system, revealing a strong linear relationship between CCW concentration and CT values (Figure S46a, Supporting Information). The CT value slope measured 53.8 HU L g^−1^, surpassing iohexol by 1.4 factor (37.8 HU L g^−1^). Moreover, clear tumor visualization was achieved post‐injection of HP‐CCW@LA hydrogel within the tumor site of mice. It contrasted distinctly with the control group, validating its remarkable CT imaging capabilities (Figure S46b, Supporting Information). Figure S47a (Supporting Information) illustrates that the PA signal of CCW‐NH enhances its concentration tandemly. After injecting the HP‐CCW@LA hydrogel, the PA signal at the tumor site showed marked enhancement compared to the control group (Figure S47b, Supporting Information).This outcome indicates that following tumor treatment using HP‐CCW@LA, it also possesses the potential for continuous monitoring of tumor changes. DCFH‐DA and 4‐amino‐5‐methylamino‐2′,7′‐difluorofluorescein diacetate (DAF‐FM DA) helped track ROS and NO release at the tissue level (Figure 5c). HP‐CCW@LA combined with US and laser stimulation produced the maximum amount of ROS/NO. We further investigated whether the antitumor immune responses in different treatment groups could stimulate dendritic cell (DC) maturation. Therefore, the levels of CD80 and CD86 in each group were measured by flow cytometry (Figure S48, Supporting Information). The results indicated that the CCW@LA+US+Laser group exhibited significantly higher levels of CD80 and CD86 compared to the other groups, suggesting that it effectively enhanced the tumor immunogenic response. This Immunofluorescence analysis indicated that HP‐CCW@LA combined with US and laser therapy significantly decreased FDX1 and elevated the cuproptosis effect. The HP‐CCW@LA+US+Laser group showed the highest cell damage, lowest cell proliferation, and most significant apoptosis, as assessed by H&E, Ki67, and TUNEL staining.

## 3 Conclusion

In summary, this study has developed a closely‐contacted CCW‐NH with a Z‐scheme structure using the “in‐situ synthesis‐post reduction” method for cuproptosis‐based cancer therapy. The CCW‐NH exhibits rapid copper ion release under the combined sonodynamic and photothermal effects, making it a promising therapeutic agent. Furthermore, the integration of CCW‐NH with a temperature‐sensitive hydrogel and L‐Arg forms an injectable hydrogel system (HP‐CCW@LA), which enables targeted tumor accumulation and sustained Cu^+^ presence, thereby enhancing therapeutic efficacy. This system also facilitates MRI‐based monitoring of treatment efficacy through copper ion conversion in the tumor microenvironment. This research advances cuproptosis therapy and offers new insights into the development of novel copper‐based therapeutic agents.

## 4 Experimental Section

### Materials

All chemicals were of analytical grade and used without further purification. Cobalt chloride (CoCl_2_·6H_2_O) was purchased from Tianjin Yongsheng Fine Chemical Co., Ltd. Ammonium ferrous sulfate (Fe(NH_4_)_2_·(SO_4_)_2_·6H_2_O), Sodium tungstate (NaWO_4_), Poly(allylamine hydrochloride)(PAH), L‐Arg were obtained from Aladdin, China. TMB was supplied by Tianjin kemio Reagent Co., Ltd. 3‐(4,5‐dimethyl‐2‐thiazolyl)‐2,5‐diphenyl‐2‐H‐tetrazolium bromide (MTT) and DCFH‐DA, Calcein‐AM, and PI were purchased from Sigma‐Aldrich. Paraformaldehyde (4%) was obtained from Beyotime. DAPGreen (D676) autophagy detection probe and Ferro‐orange were provided by Dojindo Molecular Technologies. BODIPY 581/591‐C11 were purchased from Thermo Fisher Scientific. 4′,6‐diamidino‐2‐phenylindole (DAPI), Annexin V‐FITC & propidium iodide apoptosis detection kits, and micro‐NO content assay kits were purchased from Solarbio Science & Technology Co., Ltd. (Beijing, China).

### Characterization

The morphology of the samples was characterized using SEM (hitachi‐4800) and TEM (FEI Tecnai G2 F20). The phase composition was determined using XRD analysis (Bruker AXS D8 Advance). The chemical valence of Co, Cu, W, and O elements was analyzed using XPS (Perkin Elmer PHI 5600). Optical properties were measured using a UV‐2550 spectrophotometer.

### Synthesis of CCW Nanomaterials

0.66 g of NaWO_4_ and 0.178 g of CoCl_2_·6H_2_O were dissolved in 38 mL of deionized water. CuSO_4_·5H_2_O (0.3 mmol, 0.6 mmol, or 1.2 mmol) was dispersed into 12 mL of deionized water, and the resulting solution was dropped into the above mixed solution. After 30 min of magnetic stirring, the solution was transferred to a Teflon‐lined autoclave and subjected to hydrothermal reaction at 180 °C in an electric oven for 24 h. The product was collected by centrifugation, washed alternately with water and ethanol twice, and dried in a vacuum oven for 12 h. Subsequently, 0.33 g of the dried sample was dissolved in 50 mL of deionized water, followed by the addition of 20 mL of NaBH_4_ solution (10 mg mL^−1^). After stirring vigorously for 3 h, the product was centrifuged, freeze‐dried, and stored at 4 °C.

### Photothermal Performance Test

CCW aqueous solutions with different concentrations (0‐1 mg mL^−1^) were exposed to a 655 nm laser with a power density of 1 W cm^−2^. The solution temperature was recorded at 30 s intervals using a thermal camera for a duration of 10 min.

### Detection of ·OH

Using 3,3′,5,5′‐Tetramethylbenzidine (TMB) as a probe, the samples react with hydroxyl radicals (∙OH) to yield oxidized TMB (OxTMB). The oxidized product exhibits a characteristic absorption peak at 650 nm, allowing for the quantification of ·OH production through the corresponding absorbance measurements. The experimental design is categorized into two groups: (1) HP‐CCW+H_2_O_2_; (2) HP‐CCW@LA+H_2_O_2_, with a H_2_O_2_ concentration of 10 mM.

### Detection of GSH

A solution comprising 280 µL of CCW (0.5 mg mL^−1^) and 20 µL of 10 mM glutathione was incubated at 37 °C. After 0–6 h, the supernatant was collected. Subsequently, 2.5 mg mL^−1^ of DTNB solution was introduced into each sample and stirred for 5 min. Finally, the absorbance at 412 nm was recorded using a UV‐Vis spectrophotometer.

### Preparation of HP‐CCW@LA Injectable Hydrogels

Hyaluronic acid (HA, 0.05 g) and varying quantities of Pluronic F127 (ranging from 0 to 4 g) were dispersed in 8 mL of distilled water. Following US treatment, a clear solution was obtained. Subsequently, CCW and 75 mg of L‐Arg were dispersed in 2 mL of distilled water. This mixture was then combined with the previously prepared solution and stirred for 3 h in an ice bath at 4 °C to form an injectable hydrogel (HP‐CCW@LA), resulting in a final concentration of CCW at 0.5 mg mL^−1^.

### Cell Culture

4T1 and L929 cells were cultured in Roswell Park Memorial Institute (RPMI)‐1640 medium supplemented with 10% (v/v) FBS (cGibco), 100 U mL^−1^ penicillin, and 100 mg mL^−1^ streptomycin at 37 °C in a 5% CO_2_ humidified atmosphere.

### In Vitro Cell Cytotoxicity Assay

The cytotoxicity of CCW was assessed on 4T1 and L929 cells. 4T1 and L929 cells were seeded in 96‐well plates at a density of 5 × 10^3^ cells per well and incubated for 24 h. Cells were exposed to varying concentrations of CCW (ranging from 0 to 500 µg mL^−1^) for 24 h. The culture medium was aspirated, and the cells were washed with PBS before the addition of MTT (0.5 mg mL^−1^, 20 µL) for a 4 h incubation. Color development was initiated by adding DMSO, followed by absorbance measurement at 490 nm using an enzyme reader.

### Lysosome Localization and Lysosomal Damage

Lyso‐Tracker Red was used to visualize the intracellular localization of CCW within lysosomes. 4T1 cells were seeded in a 35 mm culture dish at a density of 1 × 10^5^. Following a 24 h incubation, the culture medium was replaced with fresh media containing FITC‐labeled CCW (250 µg mL^−1^). After 1 to 6 h of incubation, the cells were washed three times with PBS. To assess the integrity of lysosomal membranes in 4T1 cells, we conducted cell staining using AO dye. Subsequently, they were exposed to a 655 nm laser at a power density of 1.0 W cm^−2^ for 10 min. Each dish was subsequently treated with 500 µL of AO solution (20 µg mL^−1^) and incubated for an additional 20 min at 37 °C in the dark.

### Measurement of ROS

ROS generation in 4T1 cells was assessed using the DCFH‐DA probe. Briefly, 4T1 cells were seeded in 35 mm culture dishes at a density of 1 × 10^5^ cells per dish and were incubated overnight. The culture medium was then replaced with fresh medium containing CCW at a concentration of 250 µg mL^−1^, and cells were incubated for 24 h. Subsequently, cells were washed with PBS and stained with DCFH‐DA (10 µM) for 30 min. Cells were then washed with PBS and exposed to a 655 nm laser at a power density of 1.0 W cm^−2^ for 10 min. Fluorescence images were captured using a fluorescence microscope with excitation/emission wavelengths of 488 nm and 520 nm.

### Measurement of Intracellular ATP Levels

4T1 cells were seeded at a density of 2×10^5^ cells per well in 6‐well plates and incubated for 24 h. They were subsequently subjected to various treatments, including CCW, CCW+US, CCW+Laser, and CCW+US+Laser. After treatment, cells were harvested, and intracellular ATP levels were determined using an ATP assay kit following the manufacturer's instructions. The laser operated at 1.0 W cm^−2^ with a wavelength of 655 nm, while US was applied at a power density of 0.5 W cm^−2^ and a frequency of 1 MHz.

### Assessment of DNA Damage (γ‐H2AX Immunofluorescence Assay)

4T1 cells were seeded at a density of 1×10^5^ cells per well in culture dishes and incubated for 24 h. The cells were divided into five groups: control, CCW, CCW+US, CCW+Laser, and CCW+US+Laser groups. Following treatment, the cells were fixed with 4% paraformaldehyde for 10 min, blocked with BSA blocking solution, and subsequently incubated with γ‐H2AX and goat anti‐rabbit secondary antibodies at room temperature for 1 h. Cells were then washed with PBS, stained with DAPI solution for 10 min, and immediately imaged using a fluorescence microscope.

### Evaluation of DLAT Polymerization

4T1 cells (1 × 10^5^ per well) were seeded in a 6‐well plate and cultured for 24 h. Subsequently, they were co‐cultured with medium containing PBS or CCW for 6 h, followed by treatment with US or laser. The cells were then sequentially fixed with 4% paraformaldehyde, incubated with DLAT antibody overnight at 4 °C, incubated with Alexa Fluor 488 anti‐mouse secondary antibody at room temperature for 1 h, and stained with Actin‐Tracker Red for 30 min. Finally, cell staining was observed using a laser confocal microscope.

### Cell Viability Imaging and Apoptosis Assay

Cell viability was assessed using the Calcein‐AM/PI dual staining method. 4T1 cells were seeded at a density of 1×10^5^ cells per dish and cultured overnight. Cells were divided into five groups: control, CCW, CCW+US, CCW+Laser, and CCW+US+Laser. After various treatments, cells were further incubated for 6 h and stained with Calcein‐AM/PI mixed dye in the dark for 20 min at 37 °C. Subsequently, cell images for different groups were captured under a fluorescence microscope with an excitation wavelength of 488 nm. For apoptosis detection, 4T1 cells were seeded at a density of 1×10^5^ cells per well in a 6‐well plate and cultured for 24 h. Then, cells were divided into six groups and stained with annexin‐V‐FITC and PI according to the manufacturer's protocol. Flow cytometry was used for apoptosis analysis.

### Measurement of Mitochondrial Membrane Potential

4T1 cells were seeded in a 6‐well plate at a density of 1.0 × 10^5^ cells per well. After 24 h of culture, the cells were stained with JC‐1 and observed under a fluorescence microscope to assess the changes in mitochondrial membrane potential in different experimental groups.

### Western Blot Analysis

4T1 cells (2 × 10^5^ per well) were seeded in a 6‐well plate and cultured for 24 h. The cells were divided into five groups: control, CCW, CCW+US, CCW+Laser, and CCW+US+Laser. Fresh culture medium containing PBS or CCW was added to the cells, and after 12 h of incubation, the cells were treated with US (1.0 MHz, 0.5 W cm^−2^, 50% duty cycle) or laser (1.0 W cm^−2^). Total protein was extracted using RIPA lysis buffer (Beyotime). Subsequently, standard Western blotting techniques were employed to analyze the expression of several key proteins, including FDX1, LIAS, and ATP7A.

### In Vivo Antitumor Efficacy and Biocompatibility Assessment

Healthy female BALB/c mice were sourced from Vital River Laboratory Animal Technology Co., Ltd. (Beijing). A suspension of 4T1 cells mixed with extracellular matrix gel (1:1) in a volume of 200 µL was subcutaneously injected into the upper right thigh of each mouse. When the tumor volume reached ≈100 mm^3^, the mice were randomly divided into six groups, each consisting of 5 mice, for drug administration. The six experimental groups were as follows: (1) Control group; (2) HP‐CCW@LA; (3) HP‐CCW@LA+US; (4) HP‐CCW@LA+Laser; (5) HP‐CCW+US+Laser; and (6) HP‐CCW@LA+US+Laser. Concurrently, mouse body weight and tumor volume were recorded daily. Tumor volume was calculated using the formula: Volume = (Length × Width^2^)/2. At the end of the experiment, mice were euthanized using cervical dislocation, and tumors as well as major organs (heart, liver, spleen, lung, kidney) were immediately excised. Tumor samples were collected for H&E staining and immunofluorescence staining.

### Animals and Treatments

All animal experiments were reviewed and approved by the Ethics Research Committee of Harbin Normal University (No. HNUARIA2022003) and carried out according to guidelines for the care and use of experimental animals approved by the Heilongjiang Provincial Department of Science and Technology.

### Evaluation of Synergistic Effects

Based on the Bliss Independence Model, the synergy between two treatment modalities can be evaluated. According to the results of post‐treatment tumor weight in different groups, the inhibition rate of sonodynamic therapy alone is 51.21%, while that of photothermal therapy alone is 60.97%. Using the model, we can calculate the expected combined inhibition rate (*E*) as follows:

(1) E=A+B−A×B

where A represents the inhibition rate of sonodynamic therapy and B represents the inhibition rate of photothermal therapy. Substituting the values, we obtain *E* = 80.96%. In our experiments, however, the actual combined inhibition rate reached 95.12%, which is significantly higher than the expected value, indicating a synergistic effect between sonodynamic and photothermal therapies.

### Statistical Analysis

Data were presented as mean ± standard deviation (n ≥ 3). Significant differences between two means were analyzed according to two‐sided Student's t‐test with ***P < 0.001, and ****P < 0.0001 (n = 5).

## Conflict of Interest

The authors declare no conflict of interest.

## Supporting information



Supporting Information

## Data Availability

The data that support the findings of this study are available from the corresponding author upon reasonable request.
