# Supplementary material for: Synthesis of Closely‐Contacted Cu2O‐CoWO4 Nanosheet Composites for Cuproptosis Therapy to Tumors With Sonodynamic and Photothermal Assistance
Source: Adv Sci (Weinh). 2024 Nov 21;12(2):2410621. doi: 10.1002/advs.202410621 (PMC11727377; doi:10.1002/advs.202410621)
Supplement: Supplementary file 1 — Supporting Information [file ADVS-12-2410621-s001.docx]

Supporting Information

Synthesis of closely-contacted Cu_2_O-CoWO_4_ nanosheet composites for Cuproptosis Therapy to Tumors with Sonodynamic and Photothermal Assistance

Zhuoran Yang,^+[a]^ Zhuo Li,^+[b]^ Chunyu Yang,^[a]^ Li Meng, Wei Guo,^*[a,b]^ and Liqiang Jing^*[b]^

**2. Results and Discussion**


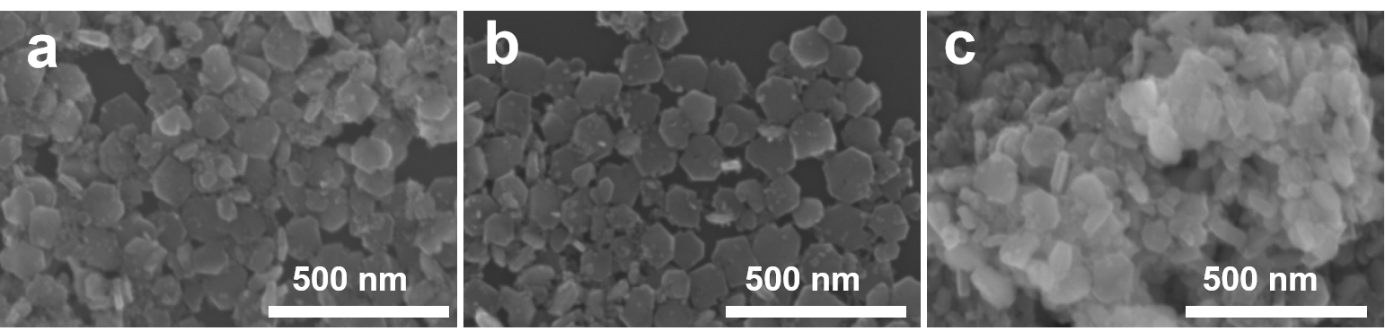


**Figure S1.** SEM images of CCW-NH precursors obtained at different reaction temperatures with a constant Cu^2+^ amount of 0.6 mmol and a reaction time of 24 h. (a) 160 °C, (b) 180 °C, and (c) 200 °C .


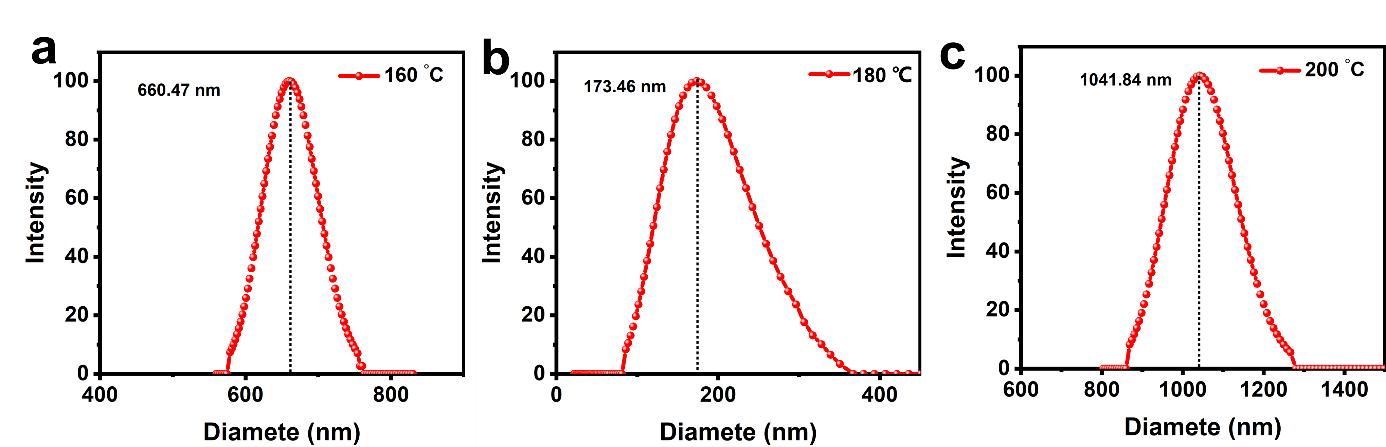


**Figure S2.** DLS of CCW-NH precursors obtained at different reaction temperatures with a constant Cu^2+^ amount of 0.6 mmol and a reaction time of 24 h. (a) 160 °C, (b) 180 °C, and (c) 200 °C.


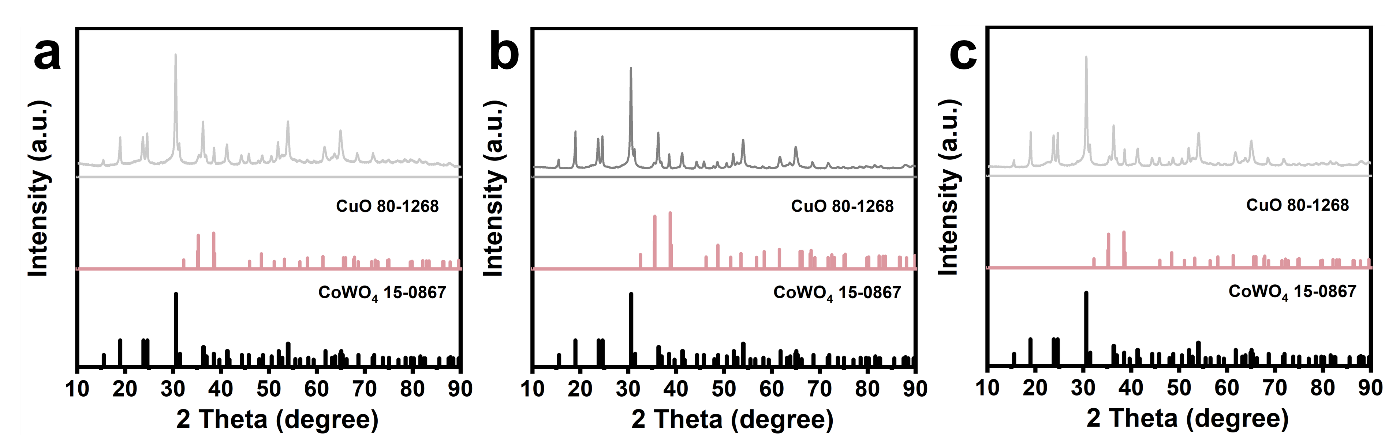


**Figure S3.** XRD patterns of CCW-NH precursors obtained at different reaction temperatures with a constant Cu^2+^ amount of 0.6 mmol and a reaction time of 24 h. (a) 160 °C, (b) 180 °C, and (c) 200 °C.


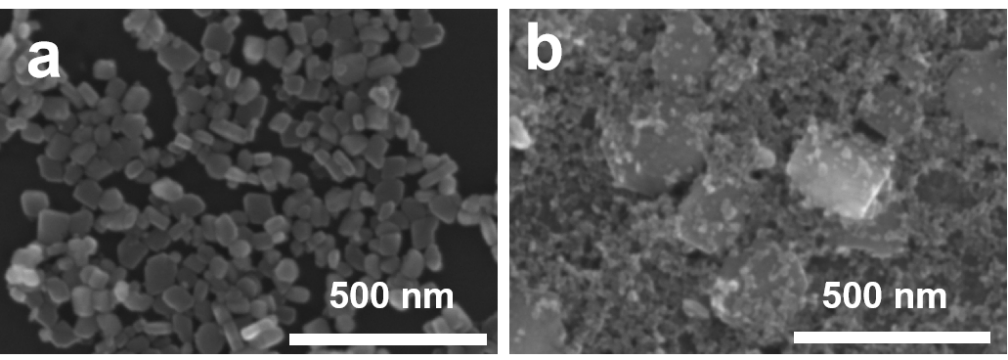


**Figure S4.** SEM images of CCW-NH precursors obtained at various Cu^2+^ amount under a constant reaction temperature of 180 °C and a reaction time of 24 h. (a) 0.3 mmol, and (b) 1.2 mmol.


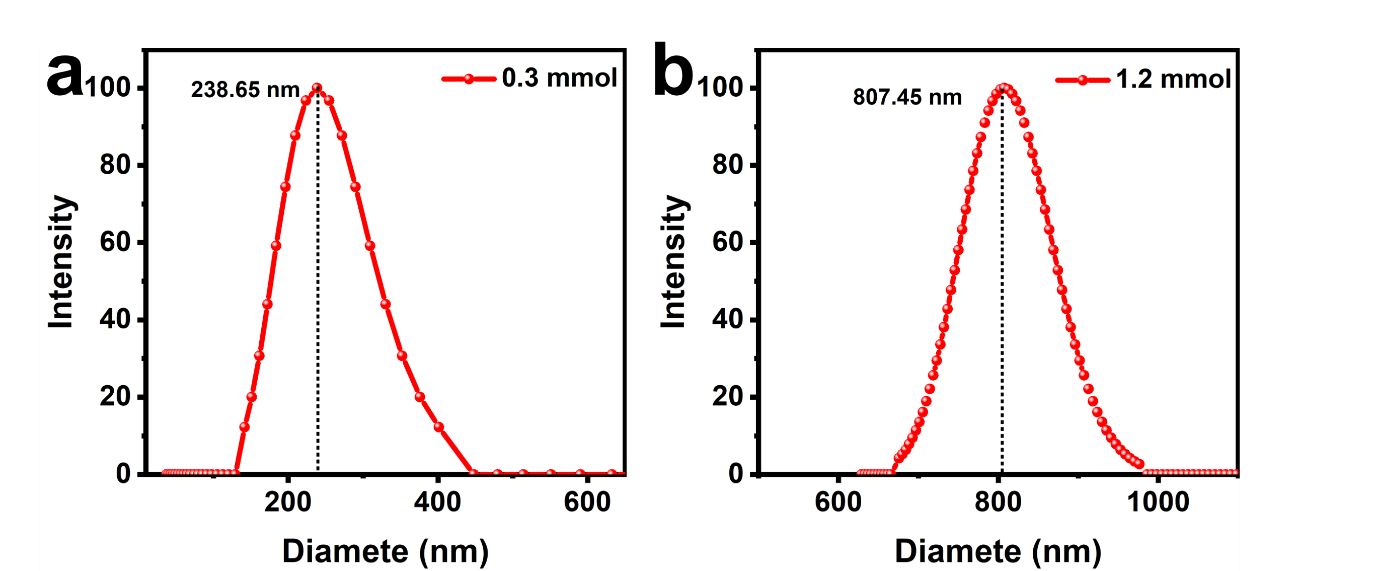


**Figure S5.** DLS of CCW-NH precursors obtained at various Cu^2+^ amount under a constant reaction temperature of 180 °C and a reaction time of 24 h. (a) 0.3 mmol, and (b) 1.2 mmol.


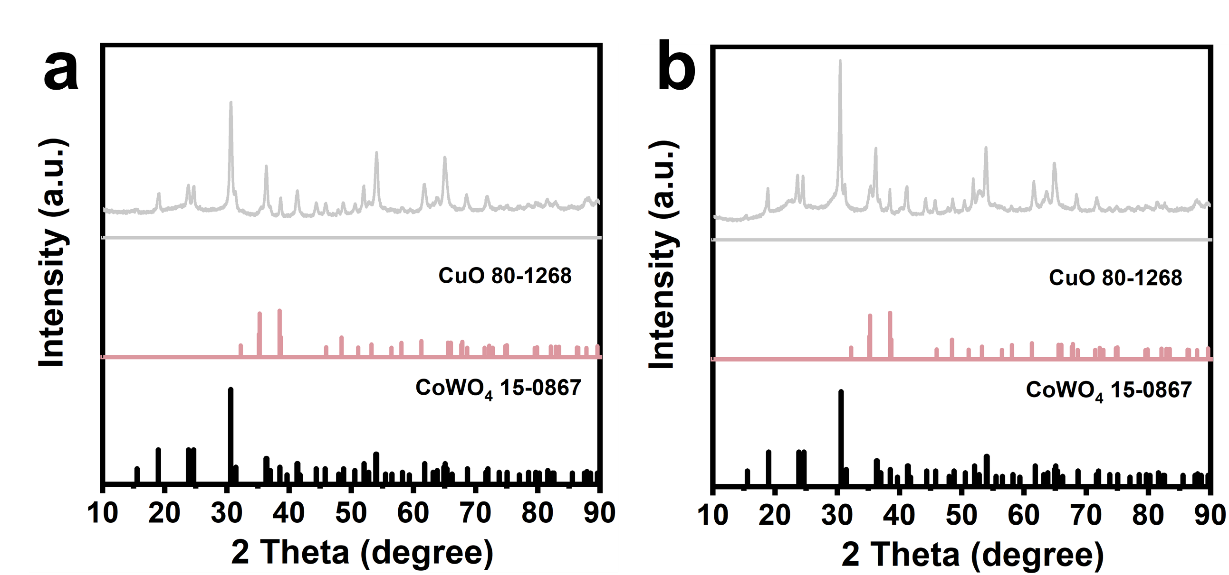


**Figure S6.** XRD patterns of CCW-NH precursors obtained at various Cu^2+^ amount under a constant reaction temperature of 180 °C and a reaction time of 24 h. (a) 0.3 mmol, and (b) 1.2 mmol.


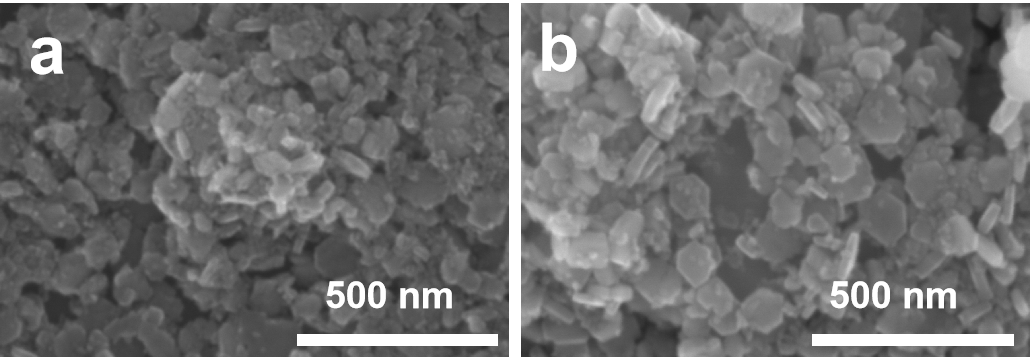


**Figure S7.** SEM images of CCW-NH precursors obtained under varied reaction times with a constant Cu^2+^ amount of 0.6 mmol and a reaction temperature of 180 °C. (a) 12 h, and (b) 36 h.


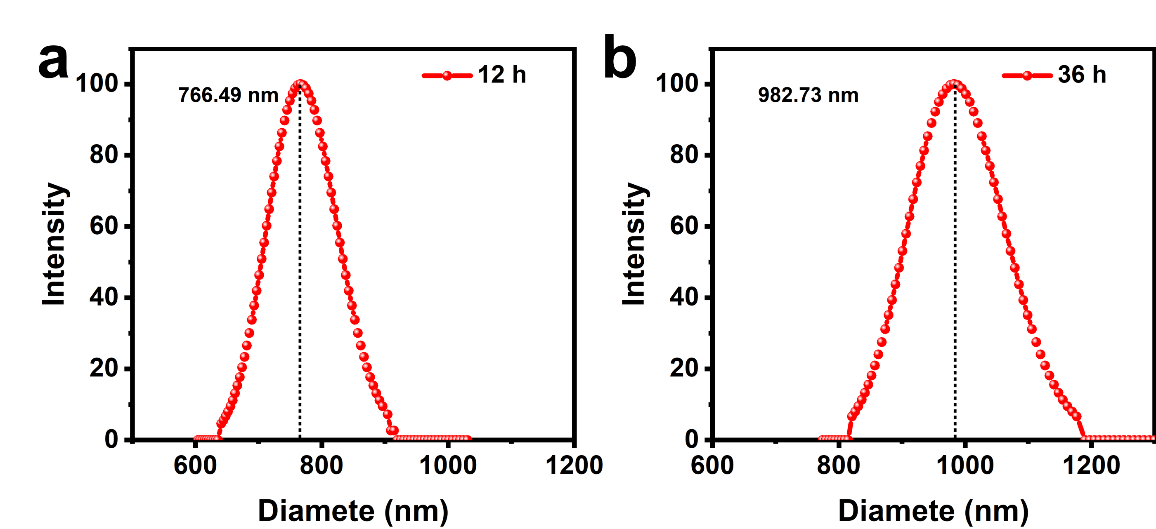


**Figure S8.** DLS of CCW-NH precursors obtained under varied reaction times with a constant Cu^2+^ amount of 0.6 mmol and a reaction temperature of 180 °C. (a) 12 h, and (b) 36 h.


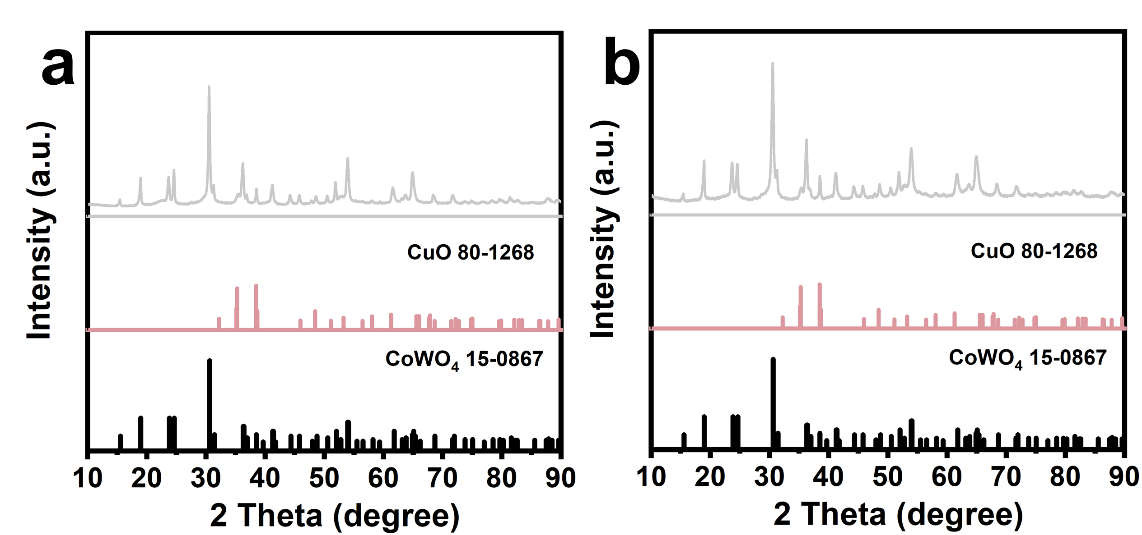


**Figure S9.** XRD patterns of CCW-NH precursors obtained under varied reaction times with a constant Cu^2+^ amount of 0.6 mmol and a reaction temperature of 180 °C. (a) 12 h, and (b) 36 h.


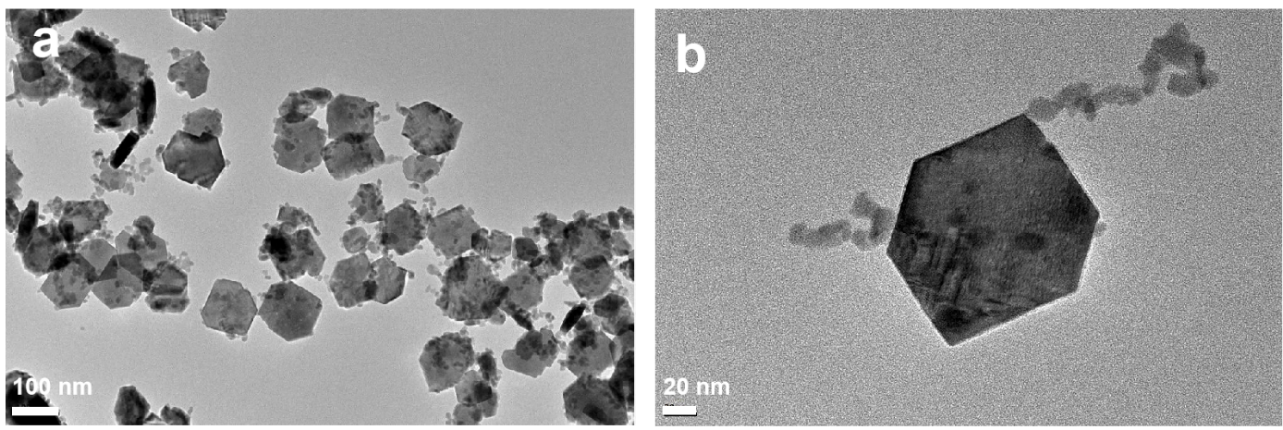


**Figure S10.** TEM images of the optimized precursor. (a) Low magnification, and (b) high magnification.


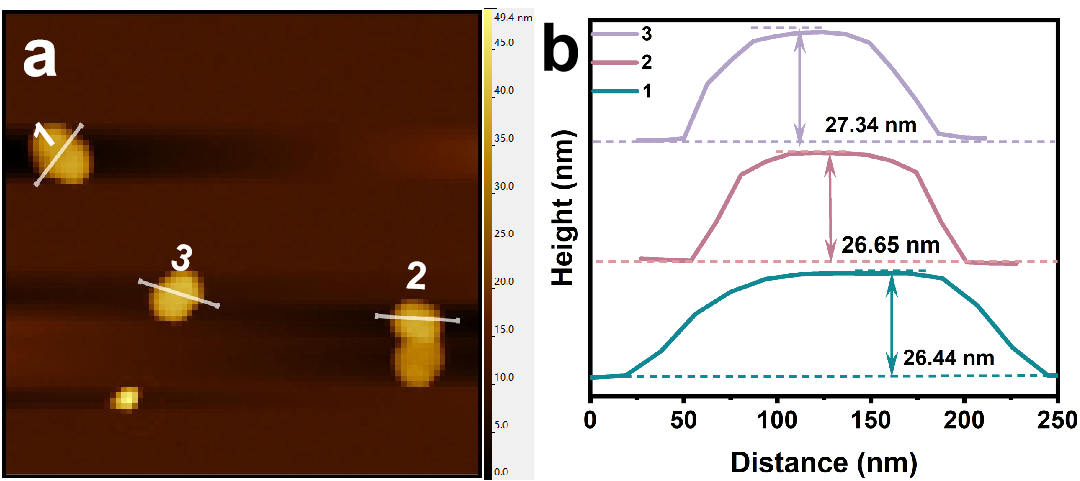


**Figure S11.** (a) AFM images and (b) the corresponding thickness measurements of the optimized precursor.


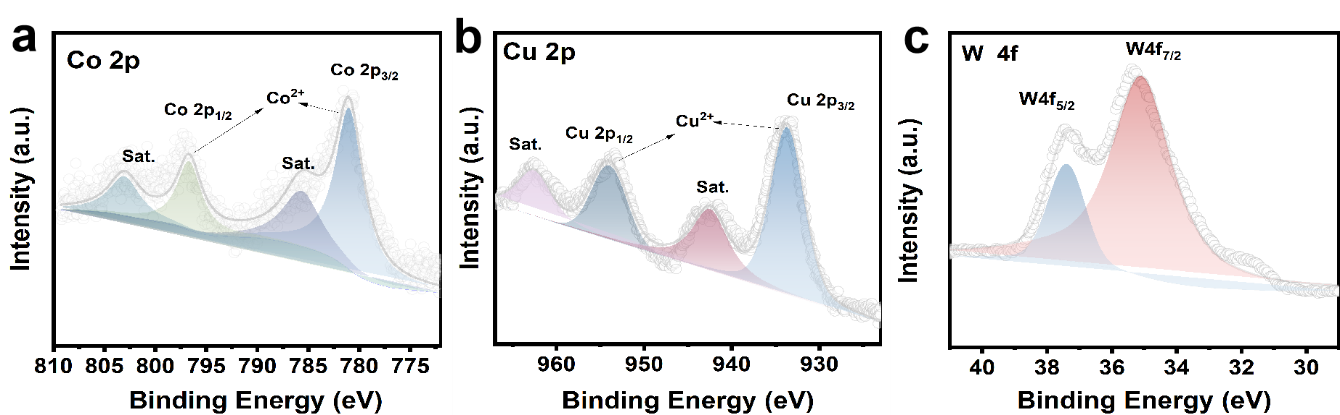


**Figure S12.** XPS of the optimized precursor.


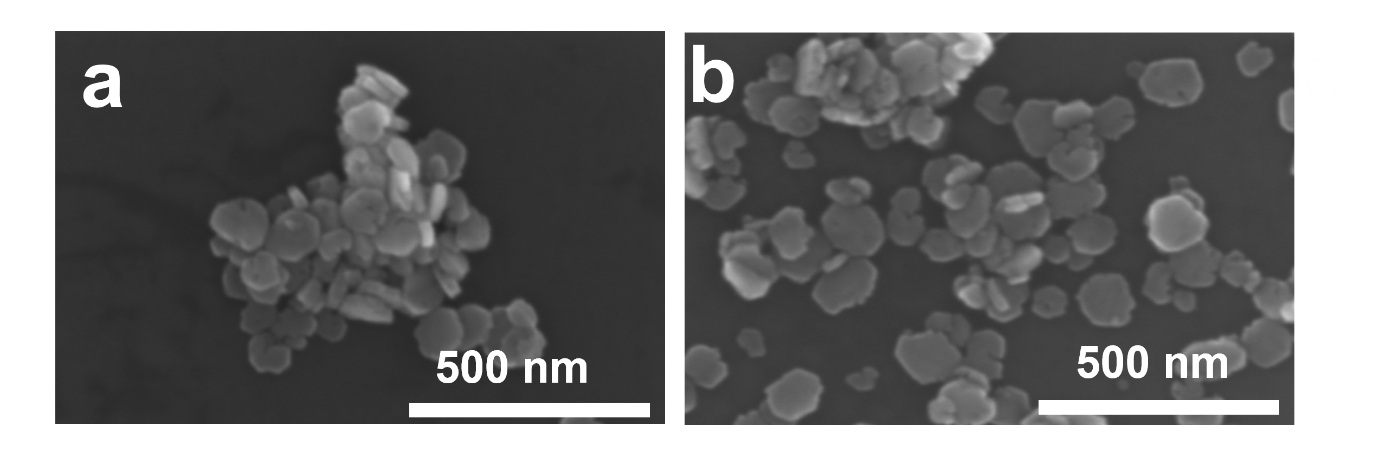


**Figure S13.** SEM images of CCW-NHs obtained by reducing the precursor with sodium borohydride for different durations. (a) 1 h and (b) 6 h.


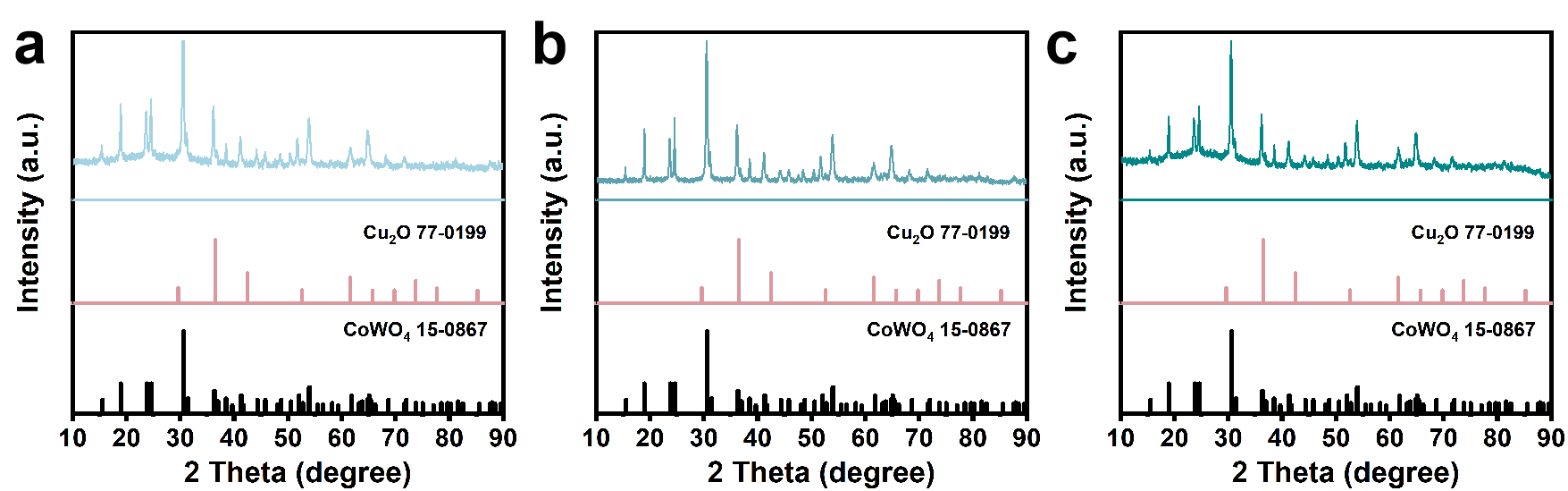


**Figure S14.** XRD patterns of CCW-NHs obtained by reducing the precursor with sodium borohydride for different durations. (a) 1 h, (b) 3 h and (c) 6 h.


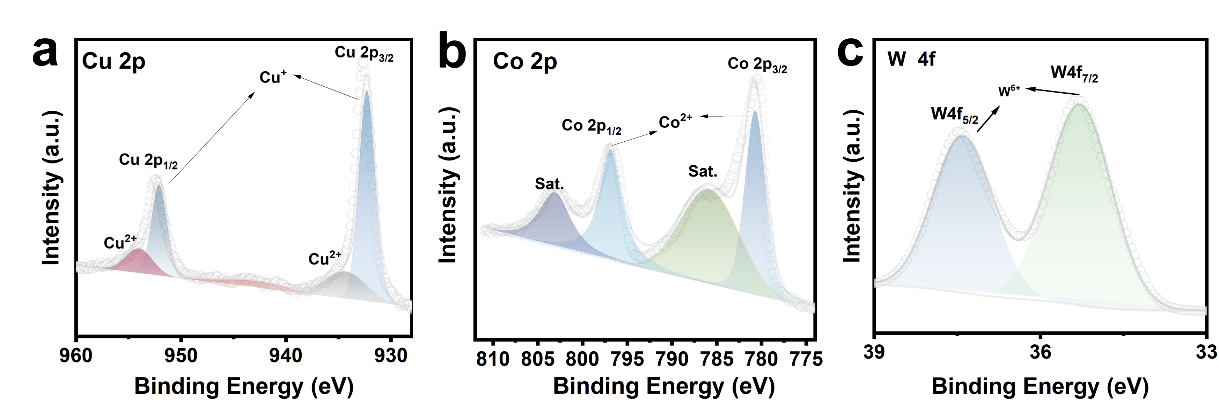


**Figure S15.** XPS of CCW-NHs obtained by reducing the precursor with sodium borohydride for 1 h.


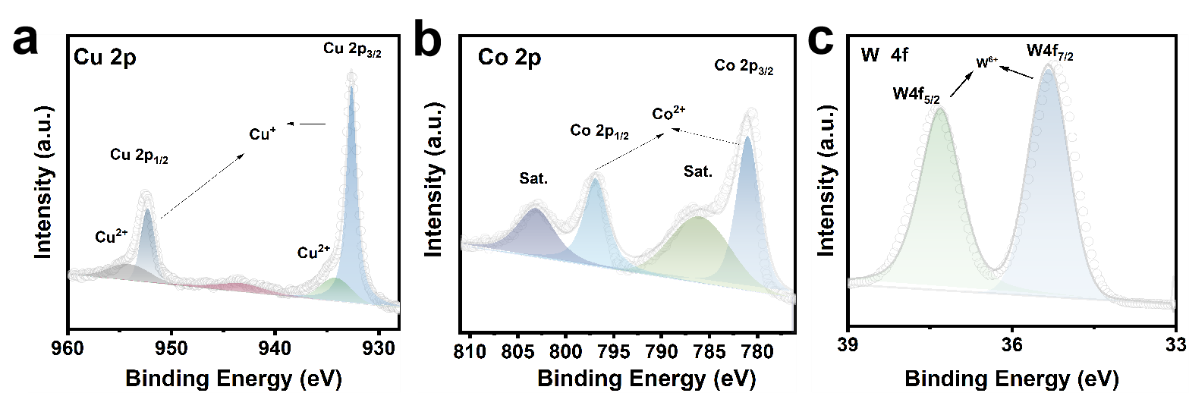


**Figure S16.** XPS of CCW-NHs obtained by reducing the precursor with sodium borohydride for 3 h.


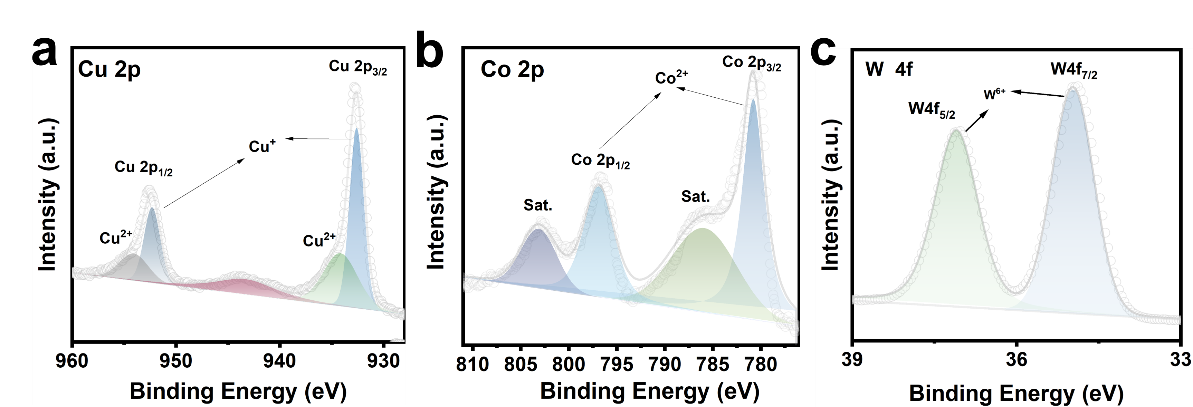


**Figure S17.** XPS of CCW-NHs obtained by reducing the precursor with sodium borohydride for 6 h.


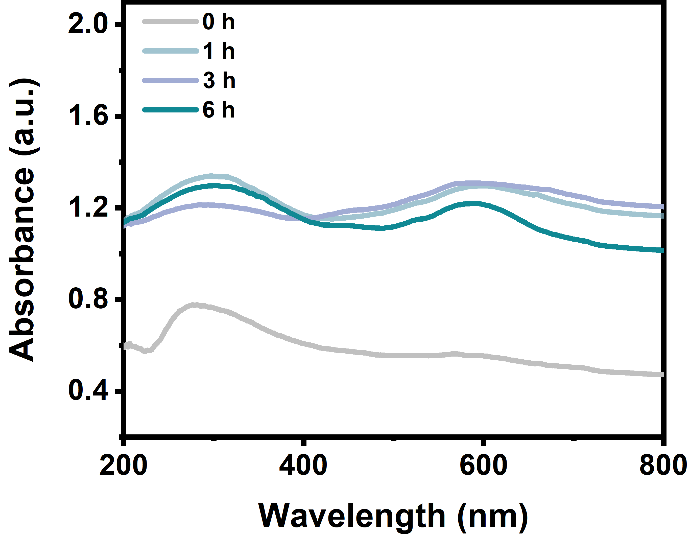


**Figure S18.** UV-vis-NIR spectra of CCW-NH with different reduction time.


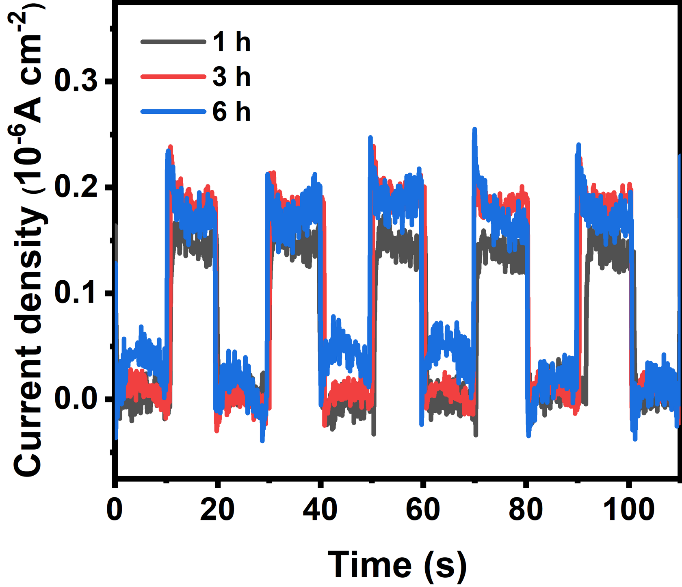


**Figure S19.** Transient photocurrent responses for CCW-NHs with different reduction time.


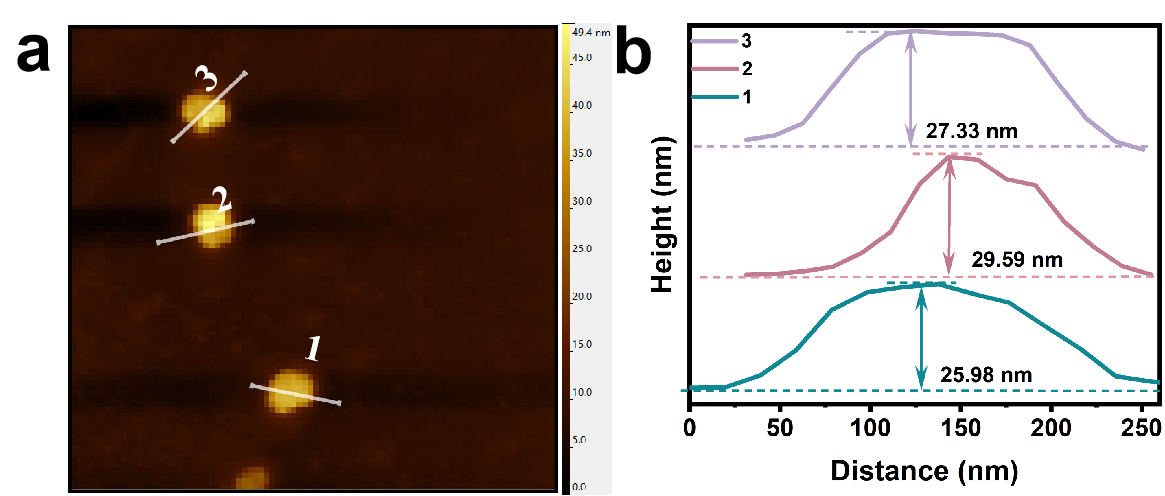


**Figure S20.** (a) AFM images and (b) the corresponding thickness measurements of CCW-NH.


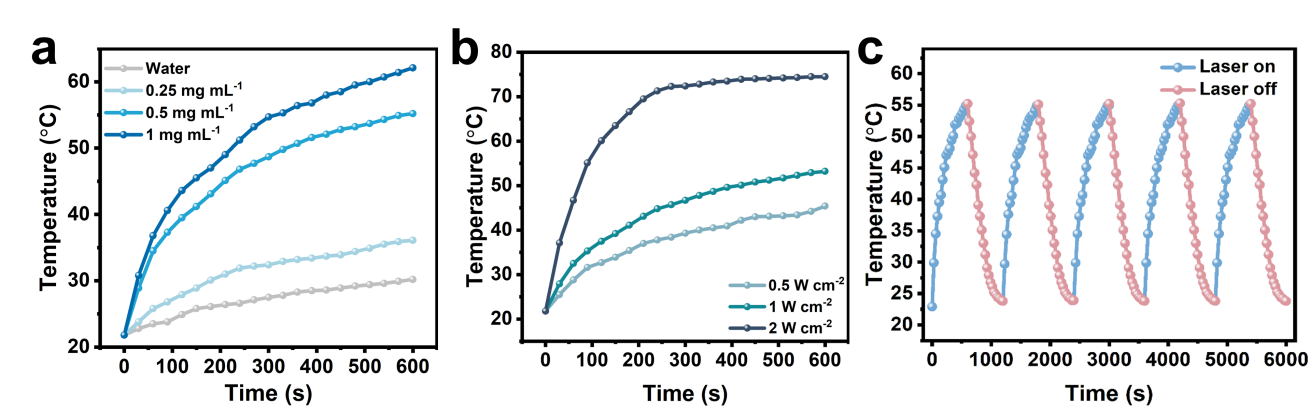


**Figure S21.** (a) concentrations and (b) power densities upon exposure to 655 nm laser radiation. (c) Results from the thermal stability test of the CCW.


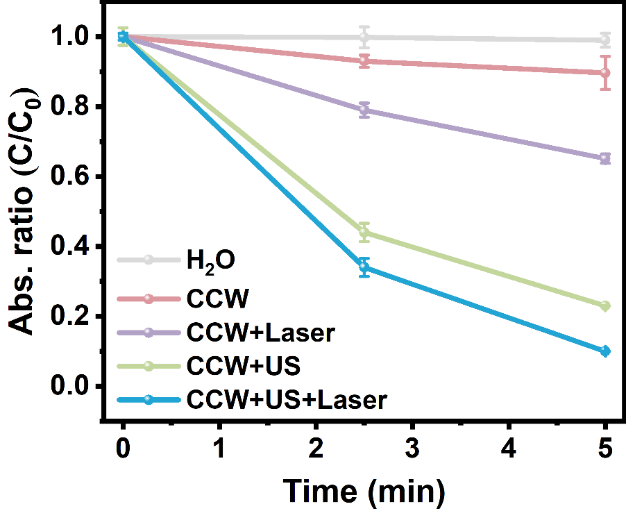


**Figure S22.** Detection of •O^2-^ using NBT probes from CCW dispersions under US, laser or US+laser irradiation. Data are presented as the mean ± SD (n = 3).


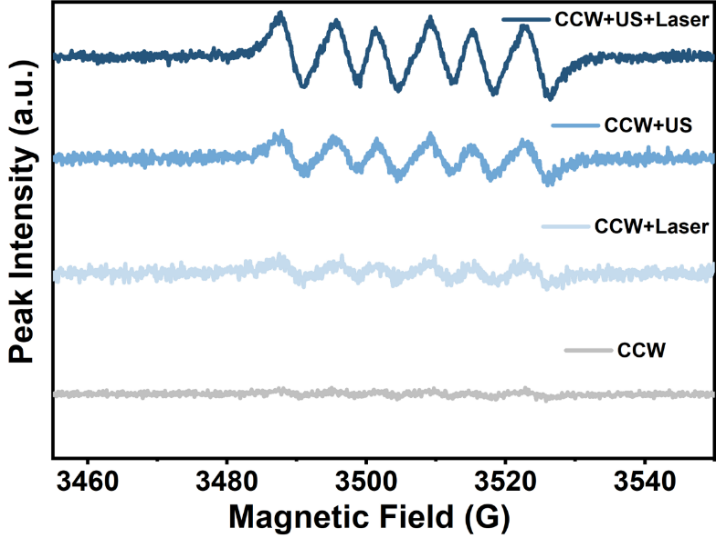


**Figure S23.** ESR spectra of different samples with DMPO probes under 10 min of irradiation.


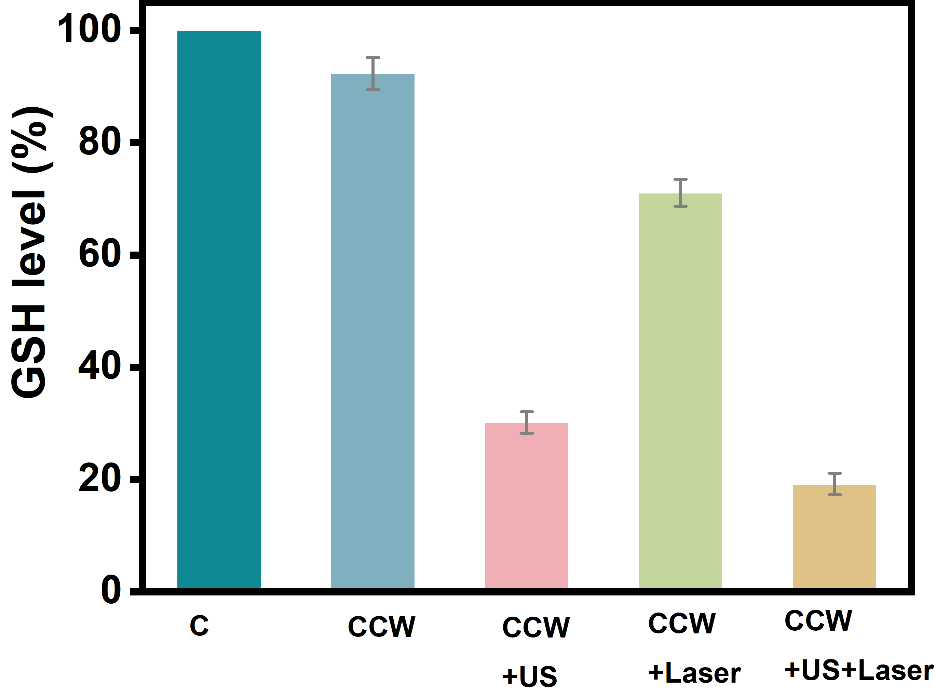


**Figure S24.** Detection of GSH using DTNB from CCW dispersions under US, laser or US+laser irradiation. Data are presented as the mean ± SD (n = 3).


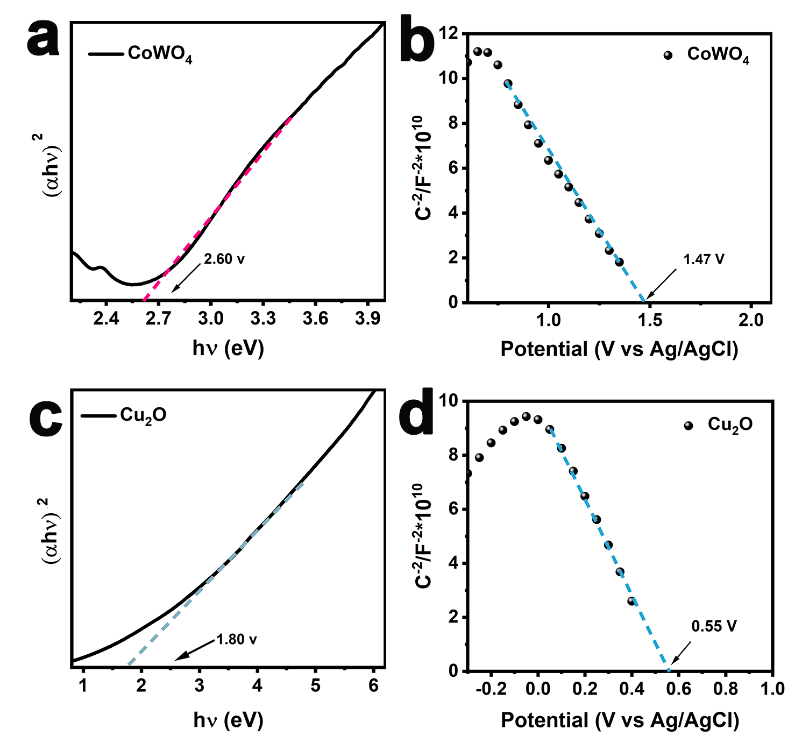


**Figure S25.** Ultraviolet-visible (UV–vis) diffuse reflectance spectra and Mott–Schottky (MS) plots for (a,b) CoWO_4_ and (c,d) Cu_2_O.


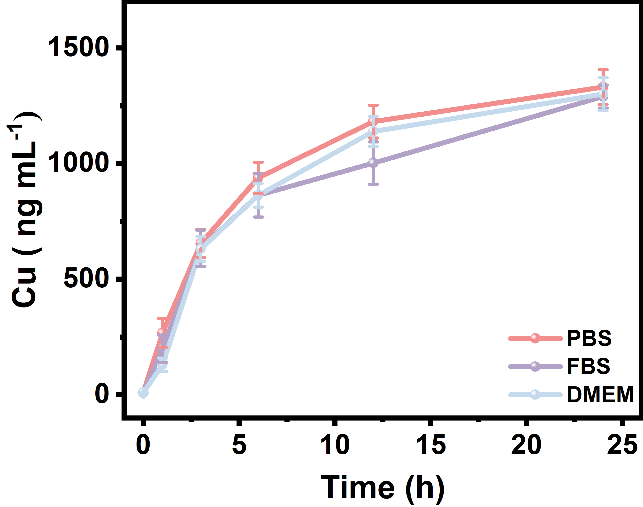


**Figure S26.** The copper ions release curves from Cu_2_O-CoWO_4_ nanosheets under different simulated physiological conditions. Data are presented as the mean ± SD (n = 3).


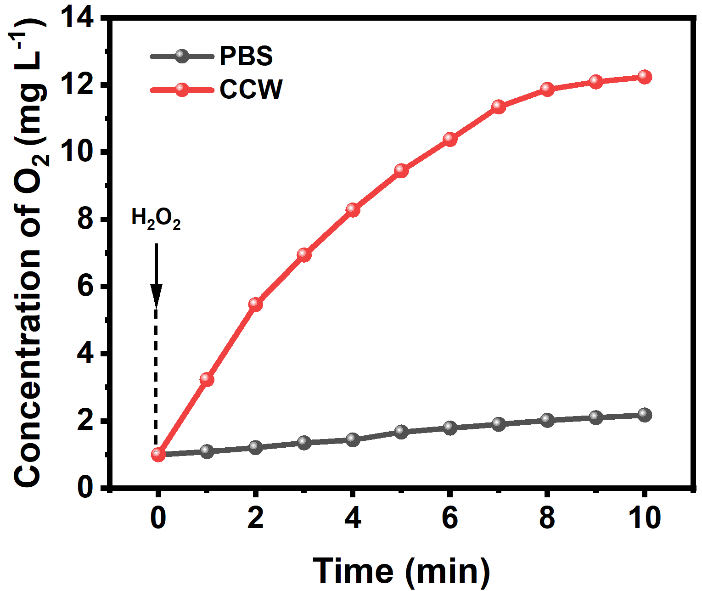


**Figure S27.** Assessment of oxygen generation by catalyzing 10 mM hydrogen peroxide using CCW solution (1 mg mL^-1^), with PBS serving as the control group.


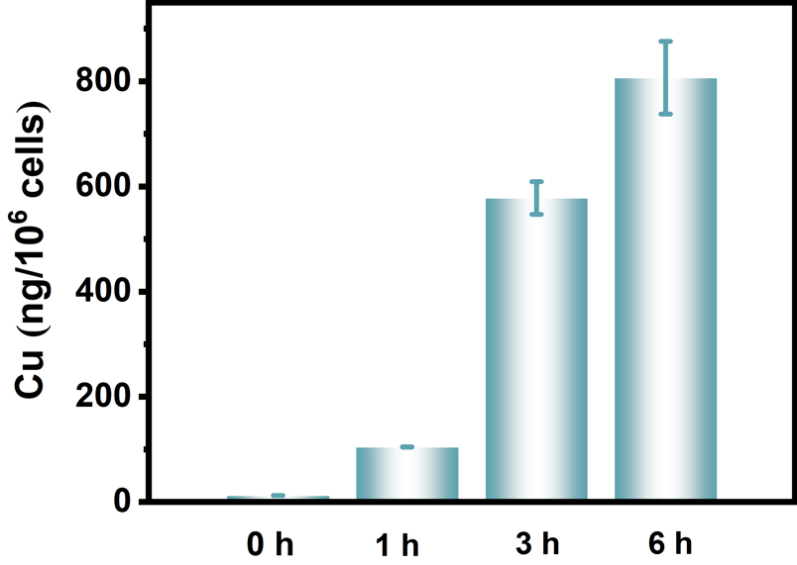


**Figure S28.** Monitoring copper release in CCW-NH co-culture with 4T1 cells over varying time intervals. Data are presented as the mean ± SD (n = 5).


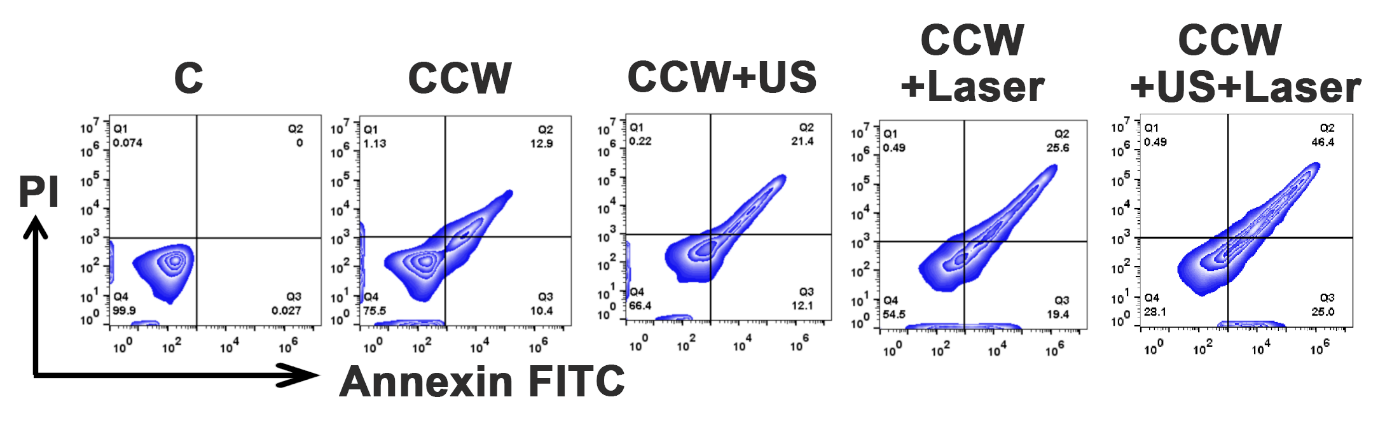


**Figure S29.** Determination of apoptotic status in diverse cell groups employing annexin V staining and flow cytometry.


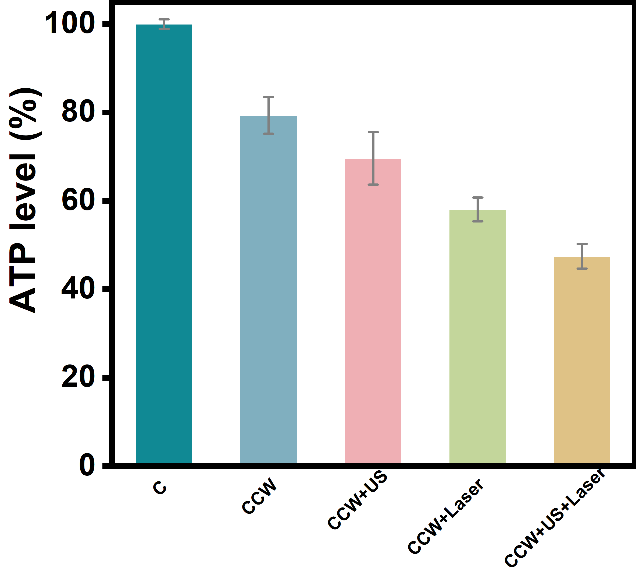


**Figure S30.** Detect the intracellular ATP content in different groups using an ATP assay kit. Data are presented as the mean ± SD (n = 5).


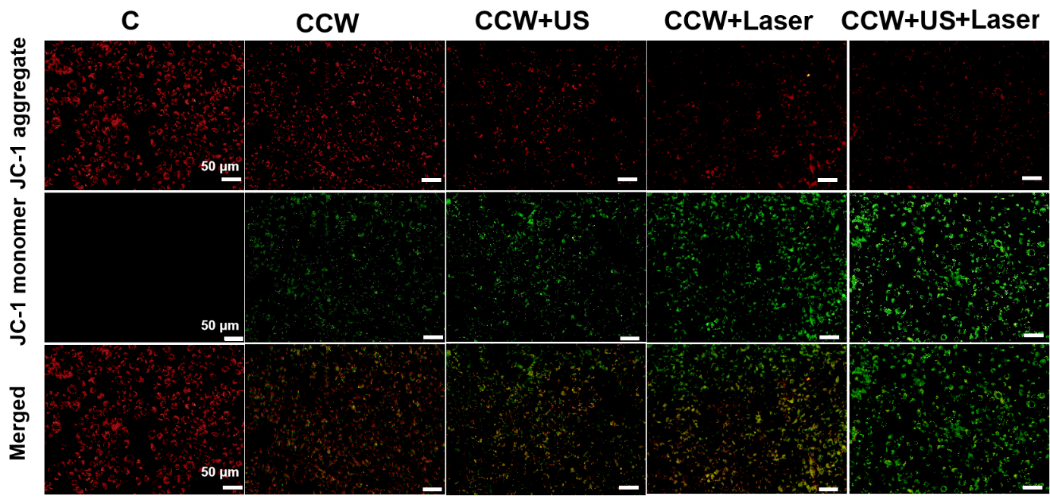


**Figure S31.** Detection of mitochondrial potential changes in different groups by JC-1 staining.


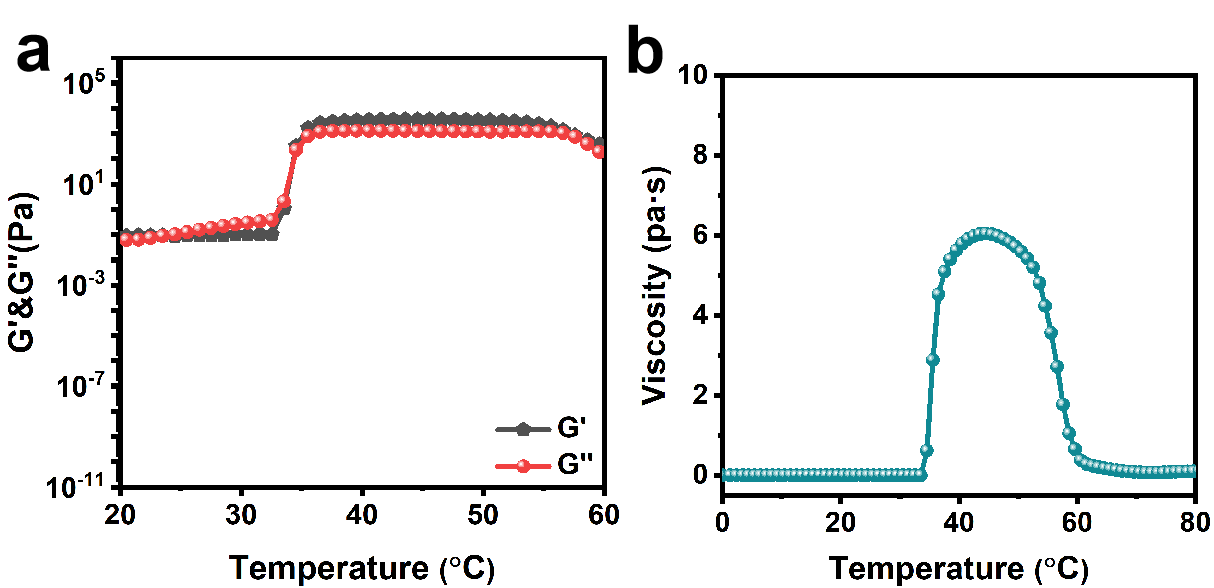


**Figure S32.** (a) Storage and loss moduli, and (b) viscosity of the HP-CCW@LA.


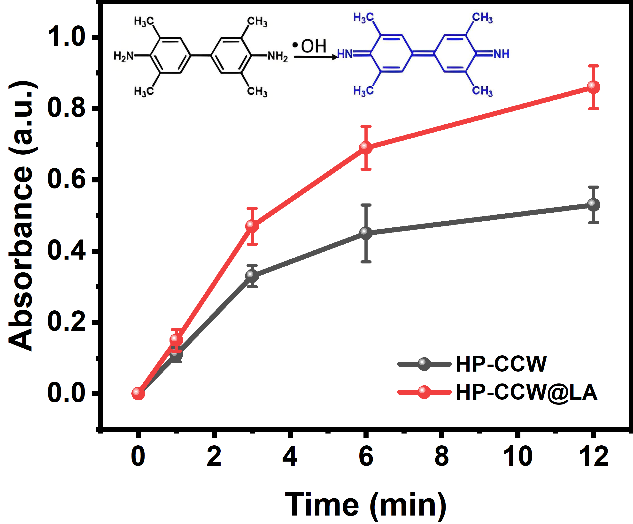


**Figure S33**. The relative TMB absorbance upon the addition of HP-CCW and HP-CCW@LA in the presence of H_2_O_2_ (10 mm). Data are presented as the mean ± SD (n = 3).


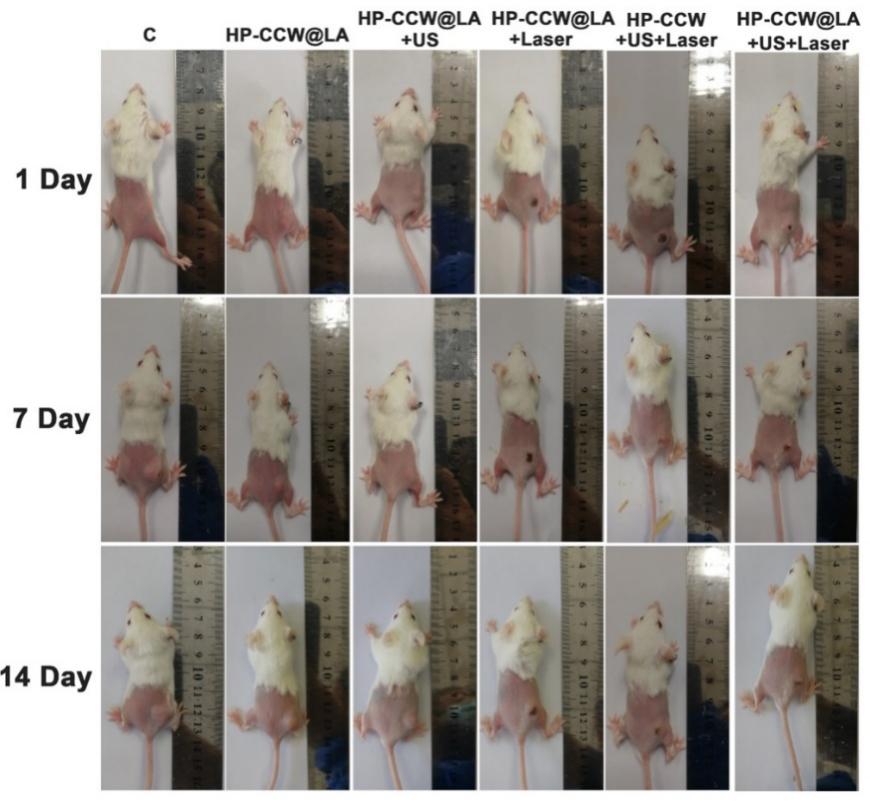


**Figure S34.** Representative photographs of mice after the 14-day treatment.


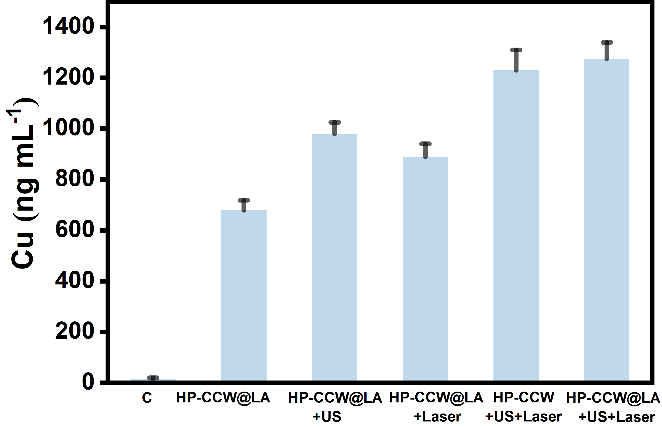


**Figure S35.** The copper ion levels within tumors after various treatments. Data are presented as the mean ± SD (n = 5).


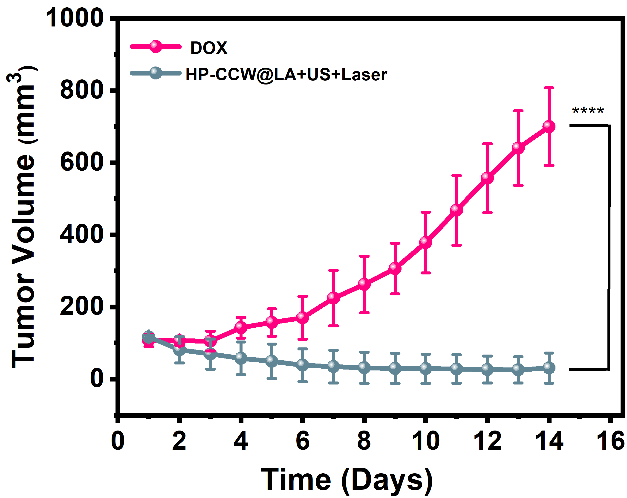


**Figure S36**. Tumor volume changes for the chemotherapy and HP-CCW@LA+US+Laser groups. Data are presented as the mean ± SD (n = 5, ****P < 0.0001).


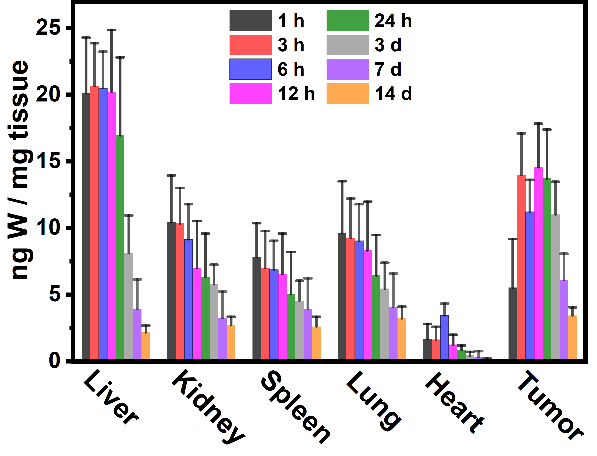


**Figure S37.** Detection of CCW biodistribution in tumors and different tissues at different time points. Data are presented as the mean ± SD (n = 5).


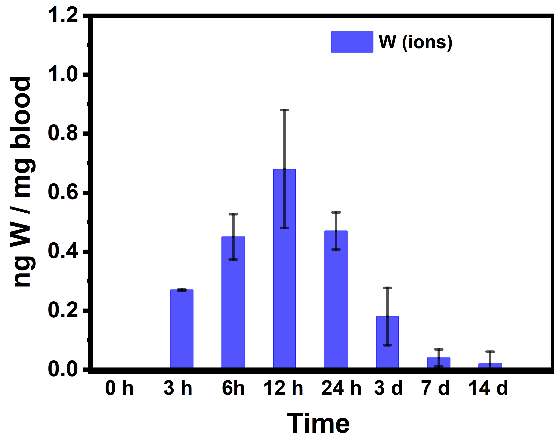


**Figure S38.** Detection of CCW-NH circulation in the blood at different time points. Data are presented as the mean ± SD (n = 5).


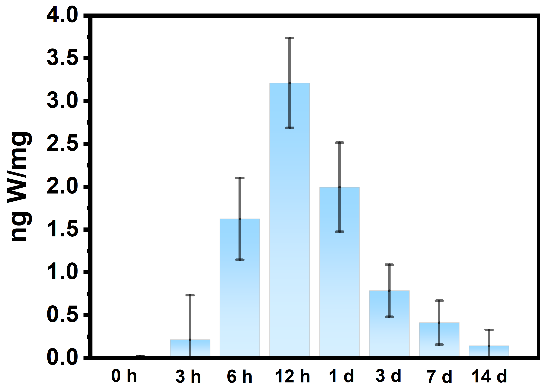


**Figure S39.** Assessment of CCW-NH clearance through changes in W element levels in urine. Data are presented as the mean ± SD (n = 5).


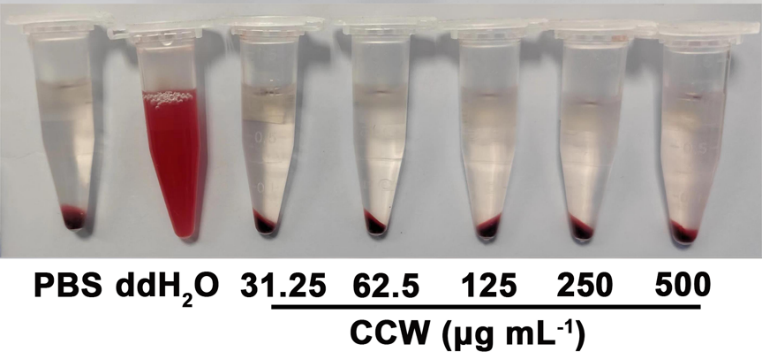


**Figure S40.** Images of hemolytic assay of red blood cells incubated with CCW at various concentrations.


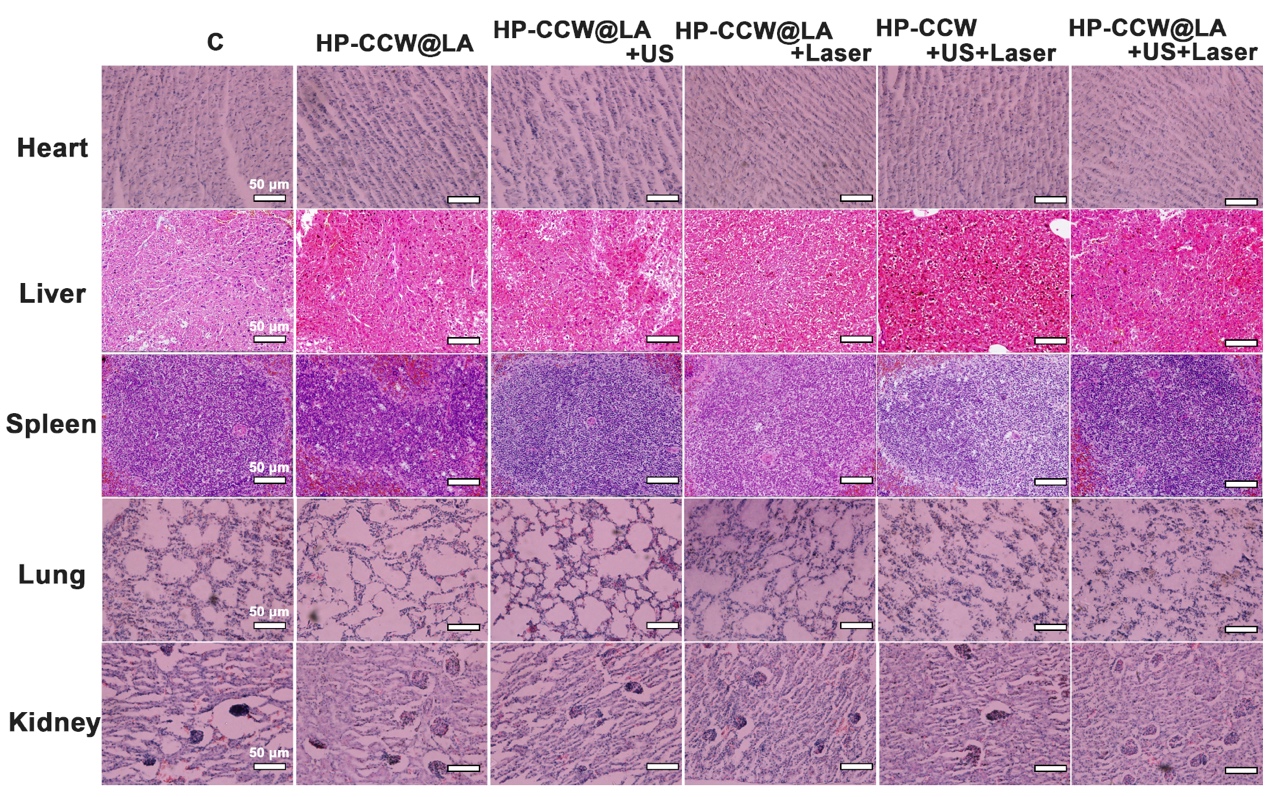


**Figure S41.** H&E-stained sections of the main organs from 4T1 tumor-bearing mice after various treatments.


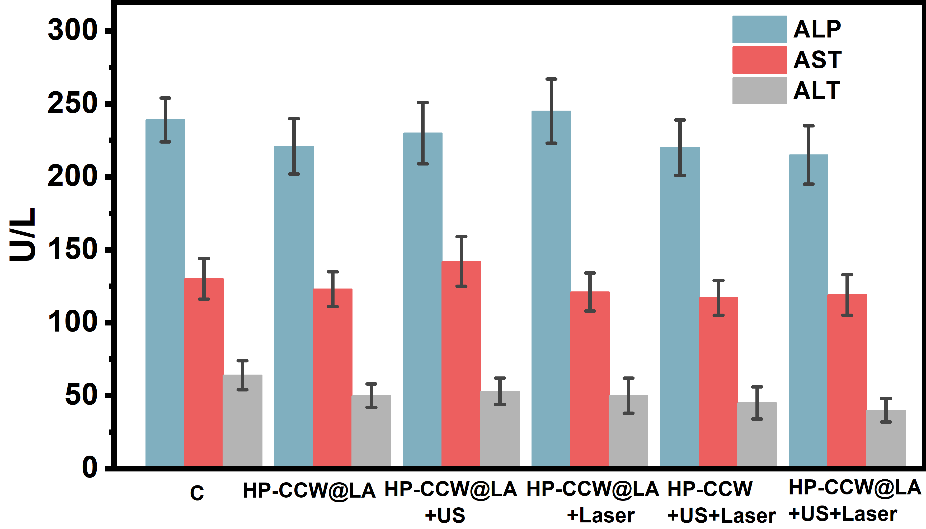


**Figure S42.** Blood biochemical data of mice after different treatments. Data are presented as the mean ± SD (n = 5).


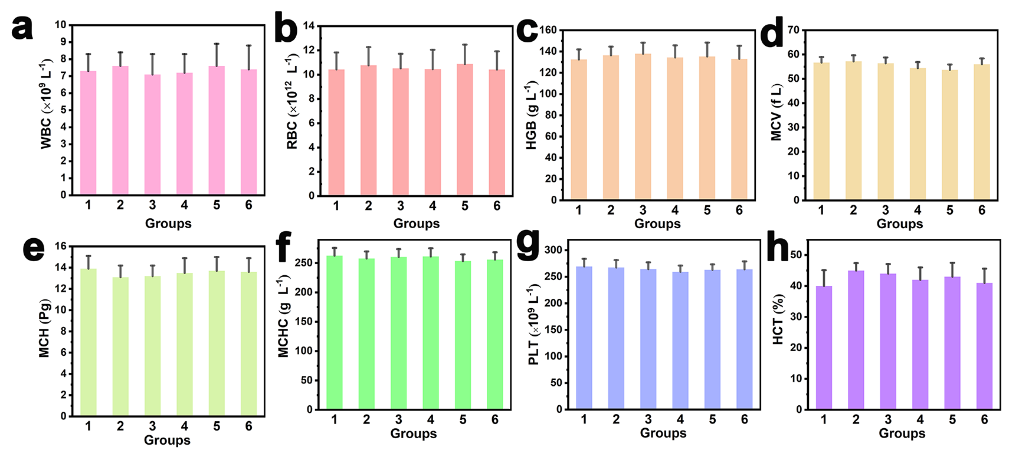


**Figure S43.** The hematology data of mice after different treatments. Data are presented as the mean ± SD (n = 5).


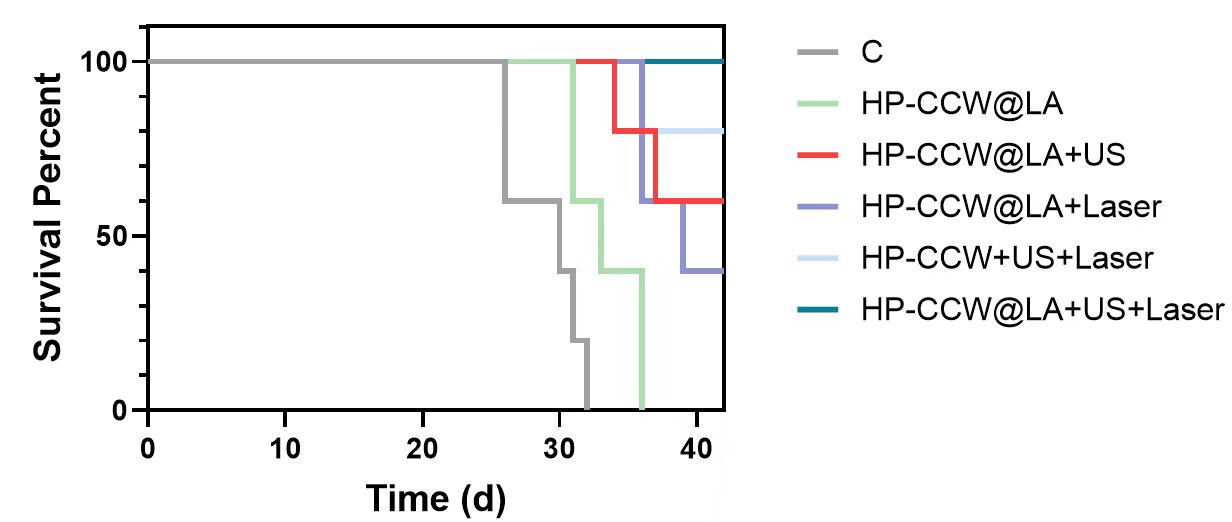


**Figure S44.** The survival curves of mice in different experimental groups.


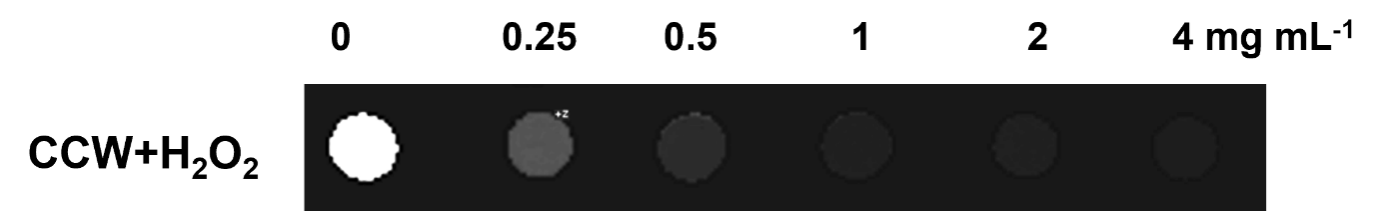


**Figure S45.** In vitro magnetic resonance imaging between concentration and 1/T_2_ for CCW+H_2_O_2_ (10 mM).


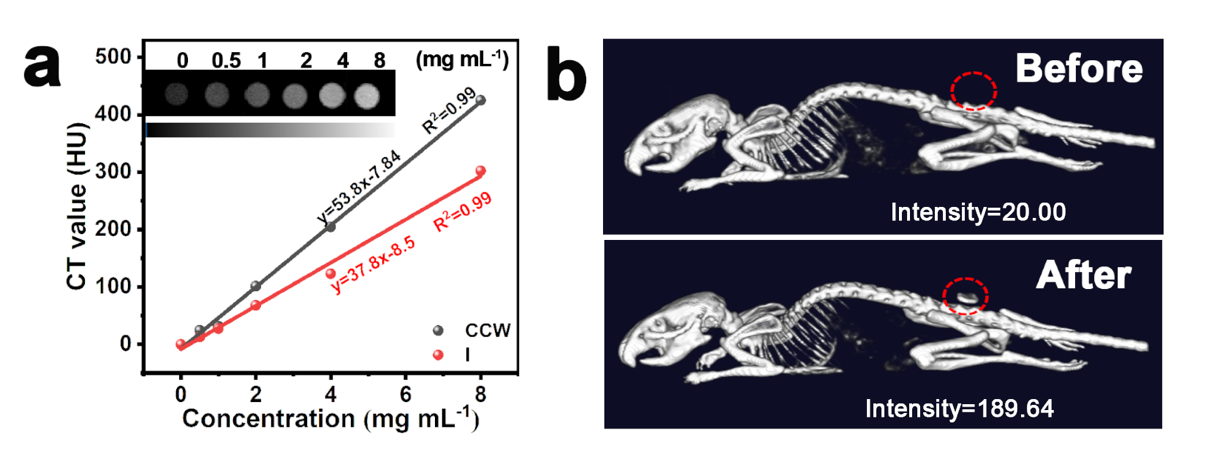


**Figure S46.** a) The relationship curve between CT value and concentration of CCW and iohexol. b) CT imaging effects of mice tumor sites before and after HP-CCW@LA injection.


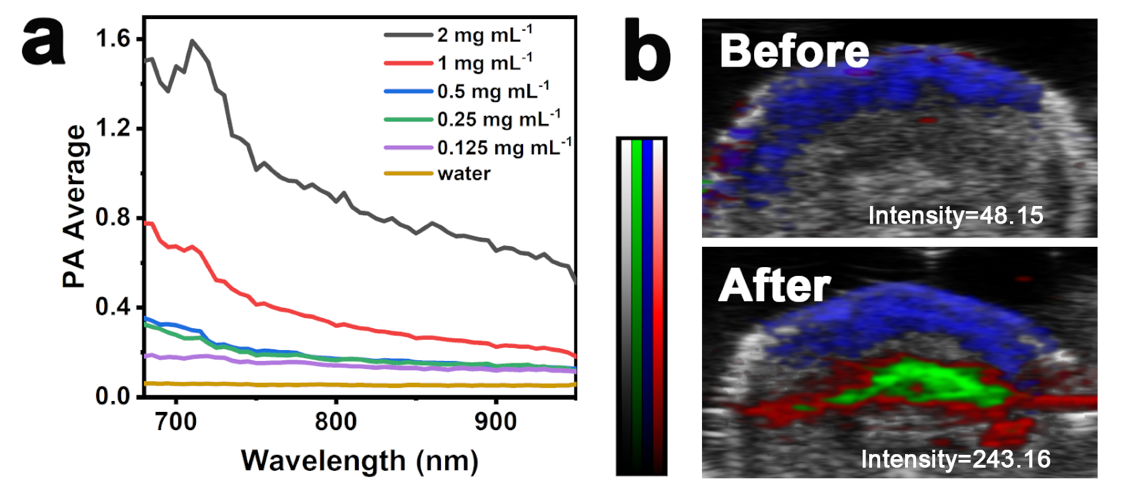


**Figure S47.** a) The relationship curve between photoacoustic signal and wavelength of different concentrations of CCW. b) PA imaging effects of mice tumor sites before and after HP-CCW@LA injection.


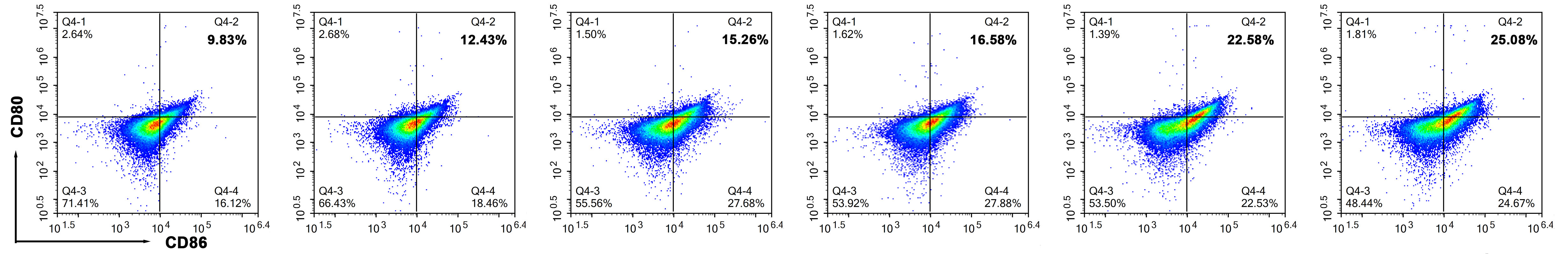


**Figure S48.** The expression of CD80 and CD86 in DCs after different treatments determined by flow cytometry.
